# How Cryo-EM Has Expanded Our Understanding of Membrane Transporters

**DOI:** 10.1124/dmd.122.001004

**Published:** 2023-08

**Authors:** Stefanie A. Baril, Tomoka Gose, John D. Schuetz

**Affiliations:** Department of Pharmacy and Pharmaceutical Sciences, St. Jude Children’s Research Hospital, Memphis, Tennessee

## Abstract

Over the past two decades, technological advances in membrane protein structural biology have provided insight into the molecular mechanisms that transporters use to move diverse substrates across the membrane. However, the plasticity of these proteins’ ligand binding pockets, which allows them to bind a range of substrates, also poses a challenge for drug development. Here we highlight the structure, function, and transport mechanism of ATP-binding cassette/solute carrier transporters that are related to several diseases and multidrug resistance: ABCB1, ABCC1, ABCG2, SLC19A1, and SLC29A1.

**SIGNIFICANCE STATEMENT:**

ATP-binding cassette transporters and solute carriers play vital roles in clinical chemotherapeutic outcomes. This paper describes the current understanding of the structure of five pharmacologically relevant transporters and how they interact with their ligands.

## Introduction

Drug transporters play a crucial role in not just the pharmacokinetic and pharmacodynamics of drugs but also in the cellular drug response to anticancer therapeutics (from the perspective of either intrinsic or acquired resistance) and in determining the response in cells that mediate other diseases (e.g., T-cells and HIV). Therefore, understanding transporter structure and function is vital for future drug development. Membrane transport proteins are necessary because drug diffusion across membranes is inversely related to the molecular weight and hydrogen-bonding capacity of the drug. Proteins that move compounds across membranes can be broadly classified as either channels or transporters. Channels form hydrophilic pores in the lipid bilayer center that permit certain solutes to rapidly traverse (up to 10 to 100 million ions/s) when the channel is open. Channels interact with their solutes through weak interactions, allowing transport to occur more quickly than with transporters (only 10–1000 molecules/s). In 1966, transporters were described in a simple model proposed by Jardetzky using three properties: 1) the protein contained a slit or interior cavity large enough to accommodate a molecule, 2) the protein assumed different conformations such that it was open on one side of the membrane and closed on the other side, and 3) the protein featured a binding site for the transported molecule, with the affinity for said molecule varying depending upon the conformation (Jardetzky, 1966). Today, transporters are also called carriers and can bind their ligands through specific interactions stronger than those used in channels. The conformational changes required for transporters to shuttle ligands cause the slower transport rate (versus channels) described previously. Predicting the binding of a ligand to a membrane transporter is a complex task that requires a detailed understanding of not only the biochemistry and structure of the transporter but also the physical and chemical properties of the ligand. Certainly, with the development of new therapeutics, many being large entities greater than 1000 g/mol the discovery of new drug transporters seems inevitable. At present, there are two major families of transporters in mammals: the ATP-binding cassette (ABC) transporters and the solute carriers (SLCs). In recent years, advances in structural biology and computational methods, such as DeepMind’s AlphaFold 2, have enabled a deeper understanding of drug transporters’ structure.

**Fig. 1.**
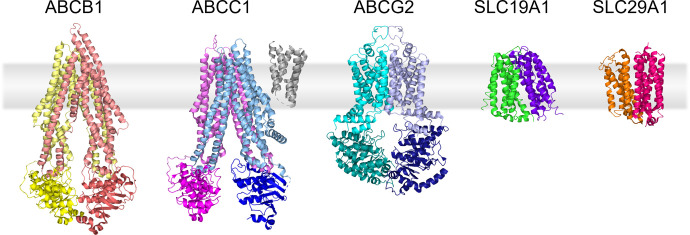
Structures of transporters discussed in the review. TMs are shown in lighter shades while NBDs are colored in darker tones. PDB accession codes are as follows: ABCB1 (6FN4), ABCC1 (5UJ9), ABCG2 (6VXF), SLC19A1 (7XPZ), SLC29A1 (6OB6).

ABC transporters are a large family of membrane transport proteins, with 48 human family members (Wilkens, 2015). ABC transporters move substrates ranging from ions to large macromolecules across lipid membranes using energy from ATP binding and hydrolysis, termed active transport (Kos and Ford, 2009; Rees et al., 2009). All functional mammalian ABC transporters contain two highly conserved nucleotide binding domains (NBDs) as well as at least two transmembrane domains (TMDs). Unlike ABC transporters, SLCs do not use ATP to move their substrates across the membrane. SLC transporters are mostly classified as either passive facilitative transporters, where the SLC moves the substrate down a concentration gradient, or secondary active transporters, where one substrate moves down its electrochemical gradient to drive transport of a second substrate against its electrochemical gradient (Pizzagalli et al., 2021). SLC transporters, with more than 400 members, are more varied in structure, with most containing 1 to 16 transmembrane helices (TMs) bundled into pseudosymmetric domains. In this review, we narrowed our focus to include only a discussion of the structures of several pharmacologically important membrane transporters and how these structures may affect their function, inhibition, and clinical outcome. The transporters discussed in this review are ABCB1, ABCC1, ABCG2, SLC19A1, and SLC29A1 because of their well-known roles in impacting response to chemotherapeutics. For reference, we have included representative Cryo-EM and X-ray crystallography structures of these transporters in Fig. 1.

### The Advent of Multidrug Resistance Transporters

Mammalian cells, when exposed to a single cytotoxic drug, often acquire resistance to multiple chemotherapeutic agents with diverse structures and mechanisms of action. This property was referred to as “multidrug resistance (MDR)”, and subsequent studies revealed that MDR was linked to reduced drug accumulation. For instance, in 1970, CHO cells cultured for actinomycin D-resistance displayed MDR to many structurally and mechanistically related drugs, including vinblastine, vincristine, and daunomycin (Biedler and Riehm, 1970). In 1973, the kinetics of daunomycin transport in a multidrug-resistant mouse Ehrlich ascites tumor cell line suggested that the resistance was associated with an energy-dependent carrier-mediated efflux mechanism (Danø, 1973). In 1976, P-glycoprotein, a surface glycoprotein, was identified from colchicine-resistant CHO cells, named because of the protein’s association with reduced drug permeability in the resistant cells (Juliano and Ling, 1976). Ten years later, the gene encoding P-glycoprotein was cloned, sequenced, and designated MDR1 (multidrug resistance protein 1) by multiple competing laboratories (Roninson et al., 1986; Ueda et al., 1986). The *Mdr1* gene has been classified as ABC subfamily B member 1, ABCB1 (human gene organism name) (Dean et al., 2001). ABCB1 is widely expressed in normal tissues, including the endothelial cells comprising the blood-brain barrier and gut epithelium. In 1992, a second ABC transporter, ABCC1, was cloned after discovery in multidrug-resistance cells not expressing ABCB1 (Cole et al., 1992; Conseil et al., 2005; Johnson and Chen, 2017). Due to the burgeoning expressed sequence database and the discovery of MDR independent of ABCB1 and ABCC1, a third ABC transporter, ABCG2, was identified by three independent groups, with their findings all published within a two-month period (Allikmets et al., 1998; Doyle et al., 1998; Miyake et al., 1999). While each laboratory reporting ABCG2 had their own noncanonical name, the common name for ABCG2 was breast cancer resistance protein (BCRP), alluding to its discovery from a drug-resistant breast cancer cell line (Doyle et al., 1998).

## ABCB1, the Original MDR Transporter

ABCB1 has broad substrate specificity and exports structurally diverse hydrophobic and amphipathic compounds including anticancer drugs, antiretrovirals, steroids, antibiotics, and *β*-blockers. ABCB1 plays an essential role as a protective physiologic barrier in several tissues, including the gastrointestinal tract, kidney, brain, testis, and placenta (Pilotto Heming et al., 2022). As ABCB1 affects the pharmacokinetics and pharmacodynamics of many drugs, the US Food and Drug Administration suggests that the interaction of novel therapies with ABCB1 is determined (US Food and Drug Administration, 2017).

To define the physiologic role of ABCB1, the gene encoding a murine ortholog of ABCB1, *Abcb1a*, was deleted and knockout mice were generated by homologous recombination (Schinkel et al., 1994). Absence of *Abcb1a* produced no obvious morphologic or physiologic defects. However, murine ABCB1 was found to be important in restricting the accumulation of ivermectin (an unknown ABCB1 substrate at the time but common antiparasitic veterinary drug) and vincristine in the brain (Schinkel et al., 1994). These findings with the *Abcb1a* knockout functionally defined a role for ABCB1 at the blood-brain barrier, previously only speculated upon based on immunohistochemical signal for ABCB1 at the blood-brain barrier (Cordon-Cardo et al., 1989). The discovery of ABCG2, the development *Abcb1a*^(−/−)^/*Abcb1b*^(−/−)^ mice, and the development of *Abcg2*^(−/−)^ mice confirmed the overlapping role of ABCB1 and ABCG2, showing synergy in restricting drug penetration at the blood-brain barrier (Wang et al., 2018). For example, brain levels of intravenously administered encorafenib were 3.4-fold higher in the *Abcb1a^(−/−)^/Abcb1b^(−/−)^* mice,1.8-fold higher in the *Abcg2^(−/−)^* mice, and 16.1-fold higher in the *Abcb1a^(−/−)^/Abcb1b^(−/−)^/Abcg2^(−/−)^* knockout mice compared with wild-type mice (Wang et al., 2018). However, the deletion of *Abcb1a/Abcb1b/Abcg2* minimally affected systemic blood levels, while brain levels of drugs affected by these transporters were markedly higher in their absence compared with wild-type mice. This difference suggested that while ABCB1 plays a crucial role at the blood-brain barrier, ABCG2 augments the barrier function (de Vries et al., 2007; George, 2016).

### Clinical Trials of ABCB1 Inhibitors

The first-generation ABCB1 inhibitors included cyclosporine A, verapamil, quinidine, and amiodarone, used to augment chemotherapy regimens (Leonard et al., 2003). The effectiveness of these first-generation inhibitors was poor for two reasons: 1) low affinity for ABCB1 and 2) exacerbation of chemotherapeutic drug toxicity. Moreover, the pharmacodynamic effect (i.e., ABCB1 inhibition) was not known in the patient samples (Gottesman et al., 2002). The second-generation inhibitors, including valspodar and dexverapamil, were more potent ABCB1 inhibitors (Leonard et al., 2003). However, these inhibitors were also limited by their off-target effects on other ABC transporters and cytochrome P450 enzymes (Leonard et al., 2002). The third generation of inhibitors, including dofequidar, zosuquidar, tariquidar, elacridar, and biricodar, were designed to inhibit ABCB1 with higher specificity, reduced toxicity, and reduced potential for pharmacological interaction. However, these third-generation inhibitors still caused inadvertent toxicity when combined with chemotherapeutics and did not show a clinical benefit (Szakács et al., 2006; Binkhathlan and Lavasanifar, 2013). Tariquidar inhibited both ABCB1 and ABCG2, which was speculated as a cause for its increased clinical toxicity (Kühnle et al., 2009; Kannan et al., 2011). Fourth-generation inhibitors have focused on natural product and phytochemical-derived ABCB1 inhibitors (Dinić et al., 2018). Many clinical trials have evaluated if adding an ABCB1 inhibitor to a chemotherapeutic regimen would improve therapeutic efficacy; however, so far, the development of ABCB1 inhibitors has been disappointing. This difficulty in adding ABCB1 inhibitors to existing regimens may stem from patients not being selected based on whether or to what extent ABCB1 is expressed in their cancer.

### ABCB1 Is a Polyspecific Transporter

**Fig. 2.**
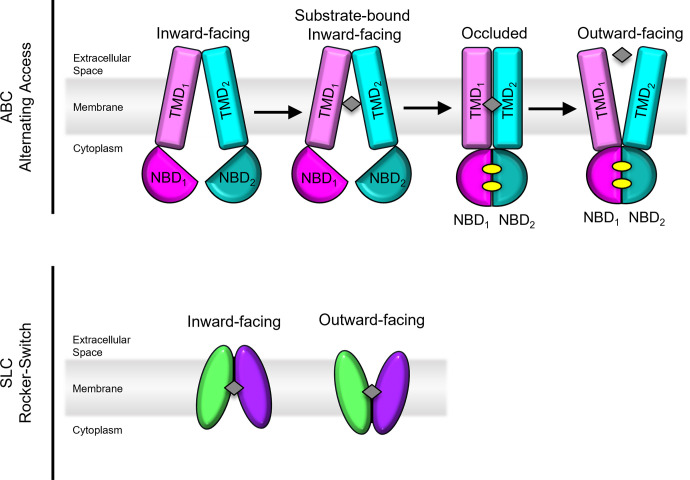
Top panel: Alternating access model of transporters used by ABCB1, ABCC1, and ABCG2. TMs are shown in lighter shades while NBDs are colored in darker tones. Substrate is denoted as a gray diamond and ATP molecules are denoted as yellow ovals. Bottom panel: Rocker-Switch model used by SLC19A1 and SLC29A1. Substrate is denoted as a gray diamond.

ABCB1 is an ∼170 KDa protein comprised of two pseudosymmetric halves connected by a linker region (Ward et al., 2013; Esser et al., 2017). Each pseudosymmetric half contains an NBD and TMD. The structure of ABCB1 has been extensively studied by multiple approaches including electron microscopy, X-ray crystallography, double electron-electron resonance (DEER), luminescence, tryptophan fluorescence, and antibody binding (Kim and Chen, 2018). Based on these studies, the “alternating access” model is the most commonly accepted mechanism of transport for ABCB1 (Fig. 2) (Ward et al., 2013). In this mechanism, binding of ATP at the NBD causes conformational changes in the TMDs, switching the transporter from inward to outward facing. During this transition, the binding cavity is open to only one side of the membrane at a time. More than 300 structurally unrelated compounds have been identified as potential ABCB1 substrates, with molecular weights ranging from 100 to 4000 Daltons (Ward et al., 2013; Kim and Chen, 2018). Most ABCB1 substrates are hydrophobic and partition into the lipid bilayer, allowing ABCB1 to pull compounds directly from the lipid bilayer to the inside of the cavity, then extruding them across the membrane. This mechanism, often referred to as a “hydrophobic vacuum cleaner,” was proposed by Raviv et al. in 1990 (Raviv et al., 1990). The binding pocket of mammalian ABCB1 orthologs contain no charged residues in the translocation pathway, in contrast to several lipid flippases (Li et al., 2014). ABCB1 can transport synthetic lipids from the inner to outer leaflets of the membrane bilayer, but there is no evidence that ABCB1 is capable of transporting physiologically relevant lipids.

### ABCB1’s Mechanism of Transport

At present, there are two slightly different proposed mechanisms of transport in ABCB1. One mechanism, proposed by Kim and Chen, was developed using the outward-facing cryo-EM structure of human ABCB1 in comparison with inward-facing structures (Kim and Chen, 2018). To stabilize the outward-facing conformation, the catalytic glutamates required for ATP hydrolysis were mutated to glutamine (E556Q in NBD1, E1201Q in NBD2 of human ABCB1). Upon NBD dimerization, the cavity observed in the inward-facing conformation changes significantly (Kim and Chen, 2018). The transition from the inward- to outward-facing conformation requires rigid-body movements of portions of each half of ABCB1 along with extensive rearrangements of certain TMs, while drug-binding residues on the surface of the inward-facing cavity reorient toward the extracellular matrix (Kim and Chen, 2018). The extracellular regions of TM7 and TM8 pull away from TM9 and TM12 to produce the outward-facing conformation. The extracellular portions of the TMDs have less defined electron densities and higher B-factors in the outward-facing cryo-EM structure of human ABCB1, suggesting these regions are flexible (Kim and Chen, 2018).

Although large changes are observed in ABCB1 during the transport cycle, the NBD-TMD interfaces remain relatively constant during the transition from inward to outward facing (Kim and Chen, 2018). This interface is important for propagating structural changes induced by ATP hydrolysis to substrate translocation, and analysis of similar inward-facing ABCB1 structures suggested that the NBD and intracellular portions of the TM helices move together as one unit during this transition.

Based on the outward-facing structure, which features a collapsed binding site free of substrate, Kim and Chen proposed that the substrate is released prior to ATP hydrolysis (Kim and Chen, 2018). In this model of transport, ABCB1 transitions between an inward-facing state with separated NBDs and an outward-facing state with dimerized NBDs. Flexibility surrounding the lateral gate allows substrates to enter from the hydrophobic membrane’s inner leaflet. With binding of ATP, ABCB1 isomerizes to the outward-facing state, thereby rearranging the drug-binding pocket to reduce substrate affinity. The flexibility of the TM helices near the outer leaflet allows the release of substrate and closure of the translocation pathway. In the outward-facing conformations, two ATP molecules are occluded, stabilizing the NBD dimer. ATP hydrolysis then returns the protein to the inward-facing conformation. This model takes into account differences between inward-facing structures from mouse, *Cyanidioschyzon merolae,* and *Caenorhabditis elegans* and an outward-facing, catalytically inactive mutant in human ABCB1 and so may not directly apply to the physiologic function of ABCB1 in humans.

A second model of transport comes from DEER studies in ABCB1. Spin-labeled pairs previously employed to monitor transitions from inward- to outward-facing conformations were used to determine the distance distributions of mouse ABCB1 in mixed-detergent/lipid micelles (Verhalen et al., 2017). Using the distance distributions, Verhalen et al. proposed instead that ABCB1 samples various conformations when ATP is bound, allowing the NBDs to dimerize when substrate binds the active site. The hydrolysis of one ATP molecule is adequate to close the intracellular gate (forming an “doubly occluded” conformation); however, hydrolysis of two ATP molecules is required for formation of the opening of the extracellular gate (and thereby formation of the outward-facing conformation). In this way, the outward-facing state is a short-lived conformation, resetting back to the inward-facing state after hydrolysis. Verhalen et al. also observed an intrinsic asymmetry of the nucleotide binding sites of ABCB1 via DEER, with occlusion of ATP at the nucleotide binding site of NBD2 and hydrolysis at the nucleotide binding site of NBD1. This asymmetry was eradicated by mutating the two catalytic glutamates of mouse ABCB1 to glutamine, the same type of mutations introduced by Kim and Chen (Kim and Chen, 2018) to stabilize the outward-facing conformation of human ABCB1. The differences in these models may arise largely due to inherent difference between the different ABCB1 homologs and the wild-type and mutant structures.

### ABCB1’s Drug-Binding Pocket

**Fig. 3.**
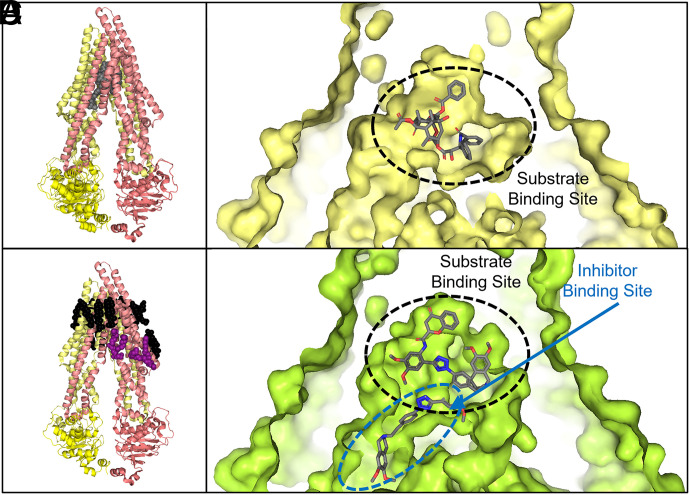
(A) Structure of ABCB1 (PDB: 6QEX). TMDs are shown in lighter shades while NBDs are shown in darker tones. Ligand binding site and access tunnel shown as gray spheres. Structure of human/mouse chimeric ABCB1 bound to Taxol, a substrate (B, PDB: 6QEX) and to Encequidar, an inhibitor (C, PDB: 7O9W). Ligands are shown as gray sticks. Substrate binding site is outlined in black dashed lines, while the inhibitor binding site is outlined in blue dashed lines. (D) Structure of human/mouse chimeric ABCB1 (PDB: 6QEX). TMDs are shown in lighter shades while NBDs are shown in darker tones. Cholesterol molecules shown in black spheres and phospholipids are shown in purple spheres. Bound Taxol molecule is not shown.

The structure of ABCB1’s binding pocket helps to explain its broad substrate spectrum. The drug-binding site, formed by ABCB1’s TM helices, is located within the cell membrane (Fig. 3A) (Li et al., 2014) Most ABCB1 substrates partition into the plasma membrane, so it is not surprising that ABCB1’s binding pocket is comprised of mostly aromatic and hydrophobic residues (Li et al., 2014). The structure of a nanodisc reconstituted human/mouse chimeric ABCB1 complexed with UIC2 Fab and one of its substrates, Taxol, exhibited an occluded conformation with a Taxol molecule bound in the central cavity (PDB: 6QEX) (Alam et al., 2019). This cavity was formed by kinks in TM4 and TM10. In this structure, Taxol-bound ABCB1 exhibits an occluded conformation with no gap between the NBDs. In this conformation, the binding pocket is formed by all 12 helices of ABCB1. While the sidechains of the binding pocket residues were well defined, Taxol was not clearly observed, suggesting that Taxol may adopt multiple binding modes. Nonetheless, due to Taxol’s size (∼854 g/mol), only a single Taxol molecule binds at a time (Fig. 3B). It should be noted that the UIC2 Fab fragment used for this structure is known to inhibit ABCB1 function by binding a discontinuous epitope on ABCB1’s extracellular loops, arresting it in the inward-facing conformation (Vahedi et al., 2018). One caveat is that this Fab-bound state might not perfectly reflect the physiologic conformation of ABCB1 with a given substrate bound; however, this approach has facilitated structural determination of other ABC transporters.

### ABCB1 Inhibition through Binding the Drug Binding Site

In several of the published inhibitor-bound ABCB1 structures, the inhibitor binds to both the substrate binding site and a second distinct site (Alam et al., 2019). The structures of human/mouse chimeric ABCB1 bound to the inhibitors elacridar, tariquidar, encequidar, and zosuquidar (along with the UIC2 Fab) all feature two molecules in the internal cavity (Fig. 3C) (Alam et al., 2018; Nosol et al., 2020; Urgaonkar et al., 2022). One molecule binds the drug binding site occupied by substrates and adopts a U-shaped, globular conformation. The aromatic rings, present in many of these ligands, position toward the top of the binding pocket, which is also rich in aromatic, hydrophobic residues (Nosol et al., 2020). For encequidar, the U-shaped molecule was lodged between the phenyl ring of F336 and F983 (Urgaonkar et al., 2022). The second inhibitor molecule adopts an L-shaped conformation and binds a distinct binding site termed the “access tunnel” by Nosol et al., located between the binding site occupied by the substrate and the kinks of TM4 and TM10 (Nosol et al., 2020). These kinks in TM4 and TM10 were found to be important for transport in *C. merolae* ABCB1, as mutation of these loops into helices diminished transport activity (Kodan et al., 2014; Kim and Chen, 2018). Kim and Chen observed that TM4 and TM10 become continuous helices in the outward-facing conformation and are necessary for full closure of the intramembranous gate (Kim and Chen, 2018).

Since these inhibitors bind the same binding pocket as the substrate, it is important to understand how ABCB1 discriminates between substrates and inhibitors. Vincristine, an ABCB1 substrate, exhibits inhibitory effects at high concentrations, while elacridar and tariquidar, ABCB1 inhibitors, may act as substrates at very low concentrations, suggesting that at low concentrations only a single inhibitor molecule occupies the substrate-binding site (Nosol et al., 2020). Nosol et al. theorized that the second, L-shaped molecule behaves more like a noncompetitive inhibitor. Substrate molecules, such as vincristine and Taxol, bound to the ABCB1-UIC2 Fab complex, are completely enclosed within the central binding pocket (Fig. 3B) (Nosol et al., 2020). Nosol et al. theorized that complete containment within this binding site allows ABCB1 to transition from inward-facing to the outward-facing state (Nosol et al., 2020). However, if the molecule cannot be contained within the drug binding site and instead occupies space in the access tunnel or connecting vestibule (Fig. 3C), transport will be inhibited. TM9 occupies part of the access tunnel and vestibule in the collapsed conformation, and TM9 acts as the peristaltic “initiator” of ABCB1’s extrusion pump (Nosol et al., 2020). Nosol et al. propose that TM9 can only complete its conformational shift when the access tunnel and vestibule are empty, as observed with substrates. When a second inhibitor molecule is bound to the vestibule or access tunnel, TM9 cannot shift due to steric clash with these compounds. In this way, the second inhibitor molecule sterically arrests the transporter in the occluded conformation, preventing the transition to the outward-facing state (Alam et al., 2019).

### PDBE-100 and the “Adaptive Plasticity” of ABCB1

Several ubiquitous pollutants found in the world’s oceans were demonstrated to be ABCB1 inhibitors (Nicklisch et al., 2016). Sixteen different chemicals were identified as ABCB1 inhibitors, 10 of which have previously been reported in humans. One pollutant, the flame retardant PDBE-1000, was crystallized inside of the mouse ABCB1 binding pocket (Nicklisch et al., 2016). The crystal structure showed that 15 amino acids from TM5-8 and TM12 participated in hydrophobic interactions with the diphenyl core of PDBE-100. The hydrophobic interactions within the binding pocket buried over 90% of the solvent-accessible surface of PDBE-1000, with the side chains adopting new conformations upon PBDE binding. Of the 15 residues identified, 9 were conserved in sea urchins, 11 were highly conserved in vertebrates, and 13 were conserved in humans (Nicklisch et al., 2016). The evolutionary conservation of these residues suggests their critical role in ABCB1 binding.

As previously mentioned, the binding pocket of ABCB1 is enriched with aromatic and hydrophobic residues. These residues account for the polyspecificity of ABCB1, since ligands are bound nonspecifically by hydrophobic amino acids instead of specific salt-bridges or hydrogen bonds (Wen et al., 2013). Aromatic residues can participate in several different interactions, including *π*-*π*, cation-*π*, and XH-*π*, with these ligands (Le et al., 2020). Due to the range of interactions available to aromatic systems, the binding pocket exhibits an “adaptive plasticity,” suggesting an induced-fit mechanism for ligands (Alam et al., 2018). When six different aromatic residues important for PDBE-100 binding were mutated in mouse ABCB1, binding remained largely intact. However, the crystal structures of these mutations showed that the binding site of PDBE-1000 shifted, with two new binding sites observed in the different ABCB1 mutants. (Le et al., 2020). The compensatory nature of ABCB1’s ligand binding suggests that this binding pocket is a challenging target for drug development (Le et al., 2020).

### Other Mechanisms of ABCB1 Inhibition

Fortunately, due to ABCB1’s extensive conformational changes required for transport, other ABCB1 inhibitors have been reported that bind outside of the binding pocket. One such compound, the nanobody Nb592, binds to mouse ABCB1’s nucleotide binding domain to hinder formation of the NBD sandwich requisite for ATP hydrolysis (Ward et al., 2013). At increasing Nb592 concentrations, ATP hydrolysis was inhibited based on reduced 8-azdio-[*^α^*^32^P]-ADP labeling in ABCB1’s catalytic sites (Ward et al., 2013). A second inhibitor, a tailored cyclic peptide, aCAP, was reported as an allosteric inhibitor of *C. merolae* ABCB1 (Kodan et al., 2014). aCAP binds ABCB1 externally, clamping down on the external helical bundle at the center of the dimer that forms the extracellular gate (Kodan et al., 2014). Binding of aCAP prevents the dissociation of the helical bundle required for transition from the inward- to outward-facing states (Kodan et al., 2014). Given the polyspecificity of the binding pocket, ABCB1 inhibition by conformational hindrance is likely a more effective strategy than targeting ABCB1’s polyspecific binding pocket.

### Structure and Function of ABCB1 NBDs

ABCB1 contains two ATPase sites, each comprising a Walker A motif and a Walker B motif from one NBD and the ABC signature sequence (Leu-Ser-Gly-Gly-Gln) from the second NBD (Kim and Chen, 2018). Despite the two active ATPase sites, only one ATP molecule can be hydrolyzed at a time, based on nucleotide trapping vanadate or beryllium fluoride (Esser et al., 2017; Kim and Chen, 2018). A highly conserved glutamate (E556 in NBD1, E1201 in NBD2 of human ABCB1) residue at the ATP site is responsible for the base-catalyzed hydrolysis of ATP. Mutating either of these glutamate residues drastically reduces ATPase activity, and both are required for full ATPase activity.

The Q-loop of ABCB1 is located at the interface between the NBDs and TMDs and has been shown to couple substrate binding to ATP hydrolysis (Zolnerciks et al., 2014; Loo and Clarke, 2017). Conserved glutamines in the Q-loop (Q475 in NBD1, Q1118 in NBD2 in human ABCB1) are important for ATPase and transport activity (Kim and Chen, 2018). Mutating these residues reduced the ATPase and transport activity and in the case of the double mutant (E to Q mutation in both NBDs) completely abolished it. Based on the human cryo-EM structure of ABCB1 in the outward-facing conformation (PDB: 6C0V), mutation of the Q-loop may destabilize the NBD dimer and prevent the molecular arrangement required for ATP hydrolysis. This structure suggests that the Q-loop is part of the interface between the NBD and TMD and may also directly contact the opposite NBD. Q475/Q1118 are located near the ATP binding sites in human ABCB1 and coordinate a Mg^2+^ ion and the *γ*-phosphate of ATP (Kim and Chen, 2018). Interestingly, the loss in activity from the double Q475A/Q1118A mutation can be restored by using a flexible cross-linker between the two TMDs, presumably bringing the two TMDs closer together to help stabilize the NBD dimer.

The A-loop of ABCB1 is a conserved aromatic residue that packs against the adenosine moiety of ATP within the nucleotide binding site (Ambudkar et al., 2006; Dastvan et al., 2019). DEER analysis in mouse ABCB1 revealed that significant changes with substrate binding were found in the distributions of spin-label pairs that monitored these A-loops (Dastvan et al., 2019). A unique short-distant component was found in the substrate-coupled vanadate-trapped post hydrolysis high-energy state (HES) at the nucleotide binding site of NBD2 but not in the equivalent spin-label pair in NBD1. This asymmetry in the nucleotide binding sites is consistent with the intrinsic catalytic asymmetry observed in the nucleotide binding sites of ABCB1. When comparing the substrate-bound and apo ABCB1 distance distributions, differences in the heterogeneity and asymmetry of the A-loops were observed. A-loop heterogeneity and asymmetry were also affected by substrate or inhibitor binding to ABCB1. Substrates that highly stimulate ABCB1’s ATPase activity induced the largest population of the short component at the nucleotide binding site of NBD2. High-affinity inhibitors, such as zosuquidar and tariquidar, induced intermediate distance components at nucleotide binding sites in both NBDs, suggesting more symmetric conformations of the A-loops with inhibitor binding. Additionally, Dastvan et al. suggested that the basal catalytic cycle of ABCB1 proceeds through a symmetric HES with reduced catalytic asymmetry. Substrate-stimulated ATP hydrolysis occurs through an asymmetric HES where one ATP molecule is occluded while the other is hydrolyzed. This asymmetry lowers the activation energy, thereby accelerating ATP hydrolysis. Conversely, inhibitors stabilize a HES that is heterogenous yet distinct from the basal cycle. The inward-facing conformation induced by inhibitor binding brings the NBDs closer together, accelerating ATP hydrolysis. However, the energy from ATP hydrolysis is not adequate to homogenously close the HES’s intracellular side to transition to the outward-facing state, which is necessary for transport.

### ABCB1’s Flexibility and Its Effect on Function

In several of the mouse ABCB1 structures, the linker connecting ABCB1’s pseudo-halves is disordered. One group attempted to stabilize ABCB1 by shortening this linker region, only to discover that mutants lacking the full linker had diminished drug-stimulated or drug-inhibited ATPase activity for compounds previously shown to act as ABCB1 substrates or inhibitors (Esser et al., 2017). Using the crystal structures of several murine ABCB1 linker mutants, Esser et al. theorized that the functional defect of the shortened linker is likely due to changes in the ligand interaction, and repeated opening and closing of ABCB1 is crucial for transport (Esser et al., 2017). By comparing these structures, it was observed that during ABCB1’s conformational transitions, TMs undergo translational, rotational, and bending movements as the molecule assumes different gap sizes between the two NBDs. Increasing NBD gap distances correlated with increasing movement in the individual TMs. TM12 was found to be particularly flexible and partially unwinds as the protein samples different conformations. Esser et al. hypothesized that, under resting conditions, murine ABCB1 undergoes nearly constant opening and closing of its two pseudohalves, which allows for the observed basal ATPase activity in the absence of substrate. The constant conformational changes of ABCB1 also produce a continuously changing architecture of the substrate-binding pocket, consistent with the “induced fit” binding mode whereby a given substrate creates its own binding site. This model is consistent with ABCB1’s vast number of substrates as well as previous inconclusive attempts to pinpoint specific binding residues for ABCB1 substrates (Bruggemann et al., 1992; Dey et al., 1997; Loo and Clarke, 2000, 2001, 2002).

### The Role of Lipid in ABCB1 Function

ABCB1’s function has been shown to be altered by the lipid membrane. Ordered cholesterol and phospholipids in ABCB1 structures suggest the membrane is vital for the conformational changes necessary for ABCB1 function (Alam et al., 2019). At the outer membrane leaflet of a mouse/human ABCB1 chimera protein complexed with UIC2 Fab, several ordered cholesterol molecules were found bound to ABCB1’s surface groove (Fig. 3D). Hydrogen bonding with hydroxyl groups on cholesterol and stacking with the R-groups of aromatic residues was observed, similar to other protein-cholesterol interactions (Alam et al., 2019). At the inner leaflet, cryo-EM density in a membrane-exposed pocket was found to be consistent with a bound phospholipid and cholesterol molecules. Binding sites for cholesterol and phospholipids are formed by kinks in the TM4 and TM10, suggesting that ABCB1 is affected by the lipids present in the cell membrane’s inner leaflet, consistent with cholesterol modulation of ABCB1 ATPase activity (Hegedüs et al., 2015; Alam et al., 2019).

## ABCC1, the Multidrug Resistance Protein

ABCC1, commonly referred to as multidrug resistance protein 1 or MRP1, is a phosphorylated glycoprotein first identified in a MDR lung cancer lacking ABCB1 (Conseil et al., 2005; Johnson and Chen, 2017). ABCC1 was later cloned in 1992. ABCC1 is frequently overexpressed in drug-selected, multidrug-resistant cancer cell lines, and *Abcc1*^−/−^ cells show tissue-specific hypersensitivity to cytotoxic xenobiotics like etoposide, vincristine, or methoxychlor. ABCC1 is normally found at blood-organ interfaces, alluding to its role in protection from xenobiotic-induced toxicity (Wijnholds et al., 1997, 1998; Cole, 2014b). ABCC1 localizes to most tissues that interact with xenobiotics, including the kidney, colon, and brain (Conseil et al., 2005; Brózik et al., 2011; Slot et al., 2011; Cole, 2014b). ABCC1 usually localizes to basolateral membranes of polarized epithelial cells (Conseil et al., 2005). In the testes, another tissue with high ABCC1 expression, ABCC1 localizes to the Leydig and Sertoli cells. ABCC1 is found in alveolar macrophages, bronchial epithelium, and hyperplastic reactive type II pneumocytes of the lung as well as the glomeruli and distal collecting tubules of the kidney. ABCC1 is found in Paneth cells of the small intestine and in the crypts of the colon. ABCC1 was identified as the essential transporter for sphingosine 1-phosphate in murine brain and spinal cord endothelial cells and was requisite for inside-out sphingosine 1-phosphate signaling to ABCB1 at the blood-brain and blood-spinal cord barriers in mice (Cartwright et al., 2013). ABCC1 may also contribute to the drug permeability barrier between the blood and cerebrospinal fluid, a possible reason for ineffective treatment of neurologic disorders (Johnson and Chen, 2017).

### ABCC1 Substrates

While ABCB1 extrudes xenobiotics, ABCC1 exports both endobiotics and xenobiotics with anionic character (Johnson and Chen, 2017). Overall, ABCC1 has numerous, structurally diverse substrates, which are typically organic anions or amphipathic organic acids with large hydrophobic groups (Johnson and Chen, 2017). Many of ABCC1’s organic anion substrates require conjugation with glutathione (GSH), glucuronic acid, or sulfate, and ABCC1 was the first mammalian ABC transporter identified to require GSH to efficiently transport some of its substrates (Cole, 2014a,b; Johnson and Chen, 2017). ABCC1 overexpression confers resistance to some anticancer drugs, both natural products and semisynthetic derivatives thereof (Zhou et al., 2008; Slot et al., 2011; Cole, 2014b). ABCC1 effluxes both hydrophobic and hydrophilic antineoplastic agents, including vincristine, doxorubicin, etoposide, and anthracyclines, thereby reducing drug accumulation within the cell and allowing for tumor resistance (Conseil et al., 2005; Cole, 2014b)_._ Antifolates such as methotrexate, flutamide, arsenical oxyanions, and antimonial oxyanions have been reported as ABCC1 substrates. ABCC1 also recognizes and transports some of the newer “targeted” anticancer agents that modify pathway components controlling tumor growth, proliferation, and metastatic potential, such as tyrosine kinase inhibitors (Brózik et al., 2011; Cole, 2014b). ABCC1 has also been found to interact with geldanamycin, which inhibits the chaperone heat shock protein 90, as well as LY294002, a reversible inhibitor of phosphoinositide 3-kinases (Abdul-Ghani et al., 2006; Pham et al., 2009; Cole, 2014b).

In addition to chemotherapeutics, ABCC1 affects the treatment of several nonmalignant diseases. ABCC1 regulates redox homeostasis, inflammation, and hormone secretion (Johnson and Chen, 2017). ABCC1 affects efficacy and disposition of several opiates, antidepressants, statins, and antibiotics and interacts with HIV inhibitors saquinavir and ritonavir (Haimeur et al., 2004; Sasabe et al., 2004; Conseil et al., 2005; Johnson and Chen, 2017)_._ ABCC1 also effluxes cysteinyl leukotriene C4 (LTC_4_), a mediator of inflammatory response. ABCC1 transports GSH, the tripeptide antioxidant, as well as glutathione disulfide. ABCC1’s transport of GSH is enhanced by phenylalkylamines like verapamil and bioflavonoids like apigenin (Leslie, Deeley, et al., 2003; Conseil et al., 2005; Leslie et al., 2005)_._ ABCC1 shares 49% sequence identity with ABCC2 and shares several substrates with ABCC2, however with differing transport kinetics (Leslie, Deeley, et al., 2003; Conseil et al., 2005; Leslie et al., 2005)_._ Probenecid and sulfinpyrazone stimulate ABCC2-mediated transport; however, these drugs inhibit ABCC1 transport activity (Bakos et al., 2000; Conseil et al., 2005).

### ABCC1 Structures

**Fig. 4.**
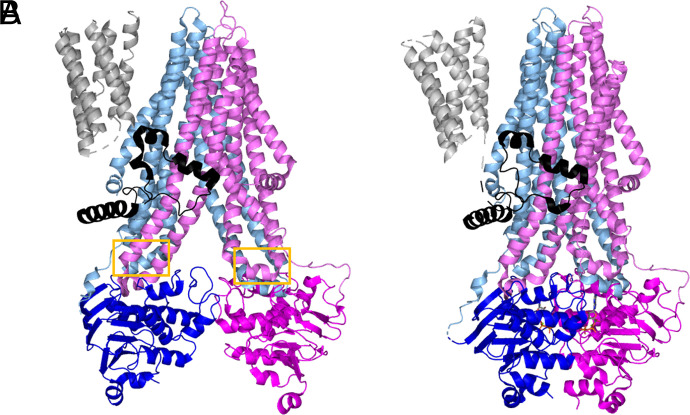
Structure of apo bABCC1 (A, PDB: 5UJ9) and ATP-bound, outward-facing bABCC1 (B, PDB: 6BHU). TMs are shown in lighter shades while NBDs are colored in darker tones. TMD0 is shown in gray and L0 is shown in black. Coupling helices are outlined in yellow boxes.

The ABCC1 gene encodes a ∼190 KDa single polypeptide that, like other ABC transporters, binds and hydrolyzes ATP to power substrate transport across the membrane (Conseil et al., 2005; Cole, 2014a; Wang et al., 2020). ABCC1 shares only 23% sequence identity with ABCB1 but contains two NBDs and two TMDs as seen with ABCB1. ABCC1’s structure differs by an additional transmembrane domain, termed the TMD0 (Fig. 4) (Conseil et al., 2005; Johnson and Chen, 2017). The TMD0 has been proposed to play a role in dimerization or stable expression at the membrane, as well as protein trafficking, regulation of protein activity, or endosomal recycling (Conseil et al., 2005; Ford et al., 2020; Bickers et al., 2021). Additional theories include that the TMD0 mediates interactions between the transporter and other protein partners; however, no protein partners have been reported, and a definitive determination of TMD0 is needed (Johnson and Chen, 2017). Determination of the role of TMD0 is further complicated by the knowledge that the role of the TMD0 may also change depending on the cell type in which the TMD0 is expressed (Cole, 2014a).

The TMs of ABCC1 contain a higher percentage of polar amino acids than the corresponding TMDs in ABCB1 (Cole, 2014a). ABCC1’s TMs contain a high number of ionizable amino acids, which is unexpected given their energetic unfavorability within the membrane bilayer (Haimeur et al., 2002; Cole, 2014a). The H-bonding capacity of these TM residues in ABCC1 shows their vital importance in substrate binding (K.I. Ito et al., 2001; Haimeur et al., 2002; Karwatsky et al., 2003; Deeley and Cole, 2006; Zhang et al., 2006; Cole, 2014a). Other transporters of organic anions feature amphipathic TM helices like ABCC1; however, these helices are considerably less amphipathic in ABCB1, consistent with ABCB1’s inability to transport organic anions (Seelig et al., 2000; Crowley et al., 2010; Cole, 2014a).

The NBDs of ABCC1 are functionally distinct from each other (Conseil et al., 2005). NBD1 exhibits a higher affinity for ATP; however, NBD2 displays higher ATPase activity (Conseil et al., 2005). Cooperativity between the two NBDs is required for ABC protein activity, and so only one ATP molecule is hydrolyzed when two ATP molecules are bound (Conseil et al., 2005.) This NBD asymmetry is unique to ABCC proteins and some other heterodimeric transporters (Cole, 2014a).

The majority of structural information for ABCC1 comes from X-ray crystal and cryo-EM structures of bovine ABCC1 (bABCC1), which shares 91% sequence identity with human ABCC1. bABCC1 conferred resistance to vincristine, actinomycin D, and vinblastine like human ABCC1 when expressed in human carcinoma cells (Taguchi et al., 2002; Johnson and Chen, 2017, 2018; Wang et al., 2020). Although the first 203 residues of the TMD0 were removed to facilitate crystallization, the functional properties of the ΔTMD0 construct, such as maximal basal turnover rate, substrate stimulation, and K_m_, were comparable to the full-length protein (Johnson and Chen, 2017). bABCC1’s structure features two TMDs arranged into pseudo-symmetric bundles (Johnson and Chen, 2017). The interface of the helical bundles forms a large transmembrane vestibule that opens to the cytoplasm and penetrates approximately halfway into the phospholipid bilayer (Johnson and Chen, 2017). The L_0_ linker immediately following the TMD0 is required for proper ABCC1 folding and function (Bakos et al., 1998, 2000; Westlake et al., 2003; Johnson and Chen, 2017). Removal of the L_0_ or the TMD0 and L_0_ linker resulted in nonfunctional transporters. However, coexpression of the L_0_ peptide along with the ΔTMD0 core particle rescued trafficking and function of the core transporter (Bakos et al., 1998; Johnson and Chen, 2017). This finding suggested the L_0_ linker structurally interacts with the other transmembrane domains (Bakos et al., 1998, 2000; Johnson and Chen, 2017).

### The Cytoplasmic Loops of ABCC1

Although cytoplasmic loops were originally thought of as only sequences that connect the transmembrane helices, there is evidence that ABCC1’s cytoplasmic loops are involved in determination of substrate specificity, proper folding and stable expression at the plasma membrane, and the transport mechanism (Cole, 2014a). Mutation of charged amino acids to alanine in cytoplasmic loop 5 and 7 caused ABCC1 misfolding, resulting in lower levels of ABCC1 at the plasma membrane (Conseil et al., 2006, 2009; Iram and Cole, 2011, 2012; Cole, 2014a). Cytoplasmic loops located at the interfaces between the TMDs and NBDs mediate the coupling of substrate translocation through the TMDs to the catalytic ATPase activity of the NBDs (Cole, 2014a). Short alpha-helical structures occurring within these cytoplasmic loops are often referred to as “coupling helices” (Fig. 4) (Hollenstein et al., 2007; Chang, 2010; Jones and George, 2014).

### ABCC1 Binding Site is Bipartite in the Inward-Facing Conformation

Cysteinyl leukotriene LTC_4_ is a proinflammatory mediator and endobiotic substrate of ABCC1 (Leier et al., 1994; Johnson and Chen, 2017). This compound is produced and excreted by immune cells during the inflammatory response and contributes to pathologies such as asthma and anaphylaxis (Deeley et al., 2006). *Abcc1*-knockout mice transported significantly less LTC_4_ and showed an impaired response to inflammatory stimuli (Wijnholds et al., 1997). The structure of LTC_4_ contains an arachidonic acid-like moiety conjugated to GSH. LTC_4_ binds within the TMDs between the two TM helical bundles within the membrane, approximately 10 Å from the cytosol [PDB: 5UJ9 (apo) and 5UJA (LTC_4_-bound), Johnson and Chen, 2017]. LTC_4_ binds ABCC1 with submicromolar affinity, which is supported by the extensive network of hydrogen binding and Van der Waals interactions observed in LTC_4_-bound ABCC1 crystal structure (Johnson and Chen, 2017).

**Fig. 5.**
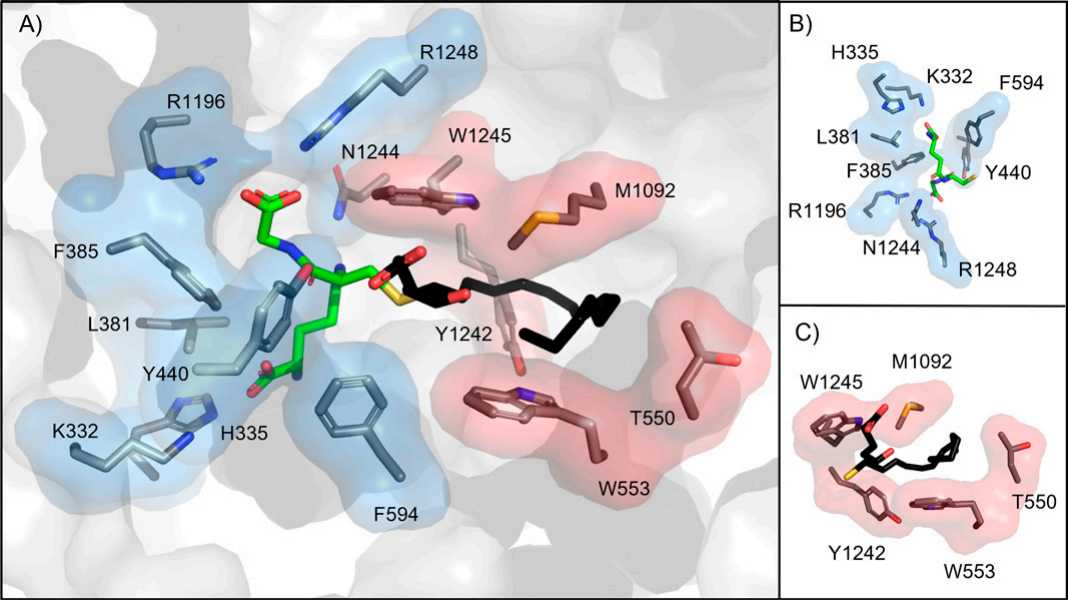
(A) Binding pocket of bABCC1 (PDB: 5UJA). P-pocket is shown in blue, H-pocket is shown in red. LTC_4_ ligand is shown as sticks, with the hydrophilic portion colored in green and the hydrophobic lipid tail colored in black. (B) Alternate view of the P-pocket only bound to the hydrophilic portion of LTC_4_. (C) Alternate view of the H-pocket only bound to the lipid tail of LTC_4_.

The binding site of ABCC1 was predicted to be bipartite in nature to allow ABCC1 to recognize large, amphipathic substrates (Loe et al., 1996; Johnson and Chen, 2017). This prediction by Loe et al. in 1996 predated the first structure of ABCC1, yet this prediction was largely correct (Johnson and Chen, 2017). The binding site of ABCC1 contains two parts: the positively charged P-pocket, which coordinates the GSH moiety of LTC_4_, and the hydrophobic H-pocket, which interacts with the lipid tail (Fig. 5A) (Johnson and Chen, 2017). Residues from both halves of the transporter form the P-pocket. In this pocket, H-bonding occurs between the GSH moiety and several residues (K332, H335, Y440, R1196, N1244, R1248; Fig. 5B). Based on other studies, K332 mutation to oppositely, neutrally, or similarly charged amino acid residue completely abolishes ABCC1 binding to LTC_4_ but has no effect on binding to organic anion substrates that do not contain a GSH moiety (Haimeur et al., 2002; Wu et al., 2005; Maeno et al., 2009; Cole, 2014a). With this result, K332 was implicated in the recognition of *γ*-glutamate portion of GSH substrate moiety (Maeno et al., 2009; Cole, 2014a). A conservative substitution of the Y440 for phenylalanine reduced GSH-stimulated substrate and LTC_4_ transport, suggesting the H-bonding between GSH and Y440’s hydroxyl group is vital for ABCC1’s substrate recognition (Grant et al., 2008). GSH also forms Van der Waals contacts with nonpolar residues in the P-pocket (L381, F385, F594) (Johnson and Chen, 2017). Unlike the P-pocket, the H-pocket is exclusively formed from one of the two bundles of ABCC1. The lipid tail of LTC_4_ bends to stack between a “tryptophan sandwich” formed by W554, M1092, and W1245 (Fig. 5C).

Comparison of the LTC_4_-bound and apo structures of bABCC1 shows substrate-induced conformational changes, both globally and locally (Johnson and Chen, 2017). LTC_4_ binds at the interface of the two TM bundles, acting as a bridge to bring the transporter halves closer together. Upon binding, rigid body rotations of the two halves (L_0_, TM bundle 1, and NBD1 form one half and TM bundle 2 and NBD2 form the other) results in the NBDs drawing closer together in the presence of LTC_4_ (Johnson and Chen, 2017). Within the binding site, the two TM helical bundles move 2-4 Å toward each other to interact with LTC_4_ while the side chains of several residues (W553, N1244, W1245, R1248) adjust to interact with LTC_4_ (Johnson and Chen, 2017). These small changes within the binding site are propagated along the transporter to the cytoplasmic NBDs, resulting in changes more than 50 Å away from the binding site. As a result, the NBDs move 12 Å closer to each other upon substrate binding and adjust their positions relative to each other, relaxing from the apo’s twisted orientation to better align the two functionally distinct ATPase sites. LTC_4_ binding stabilizes a conformation where the NBDs are closer and better aligned, allowing for dimerization to form a complete catalytic site, which partially explains the elevated ATPase activity observed for ABCC1 in the presence of LTC_4_ (Davidson et al., 1992; Oldham and Chen, 2011a).

The bABCC1 binding site residues undergo local rearrangements of their sidechains upon LTC_4_ binding, suggesting the binding site exhibits a plasticity that likely allows for the polyspecificity that ABCC1 exhibits (Johnson and Chen, 2017). In addition, the bABCC1 structure sheds light on how GSH alone can be effluxed by ABCC1 because GSH can bridge the two pockets of the transporter on its own (Paulusma et al., 1999; Renes et al., 1999; Salerno and Garnier-Suillerot, 2001; Leslie, Deeley, et al., 2003; Cole and Deeley, 2006). However, ABCC1 exhibits millimolar affinity for GSH as compared with the nanomolar affinity observed for LTC_4_. This discrepancy is caused by increased favorable interactions between LTC_4_’s lipid moiety and the H-pocket. Substrates that can be transported without the GSH moiety are likely amphipathic and can interact with both the P- and H-pockets.

### The Outward-Facing Conformation of ABCC1

To stabilize the outward- facing conformation of ABC transporters, mutations that allow for ATP binding but prevent ATP hydrolysis may be introduced into the protein (Oldham et al., 2007; Johnson and Chen, 2018). For bABCC1, the catalytic glutamate that acts as a general base in ATP hydrolysis, was mutated to glutamine (E1454Q mutation) (Oldham and Chen, 2011b; Johnson and Chen, 2018). Mutation of this residue in human ABCC1 showed decreased ATPase activity and abolished transport of LTC_4_, while showing no effect on LTC_4_ binding (Payen et al., 2003; Johnson and Chen, 2018). Cryo-EM structures of the E1454Q mutant mixed with ATP and LTC_4_ showed the two halves of ABCC1 pack closely to encompass two Mg^2+^ ions and two ATP molecules at the interface of the NBD dimers (Fig. 4B) (PDB: 6BHU; Johnson and Chen, 2018). Unlike the inward-facing conformation, the translocation pathway is closed off from the cytoplasm and opens to the extracellular space. The outward-facing conformation of bABCC1 differs from Sav1866, a prototypical eukaryotic ABC transporter. Where Sav1866s TM domains veer out like wings in the outward-facing conformation and allow the translocation pathway access to the membrane’s outer leaflet, ABCC1’s translocation pathway is closed off from the lipid bilayer, only accessible from the extracellular space.

The ATP-bound outward facing structure also sheds light on the molecular mechanisms responsible for the degenerate ATPase site of hABCC1. Where the competent site contains a glutamate residue, the degenerate site contains an aspartate (D793), whose side chain is one carbon shorter than glutamate. This aspartate likely cannot adopt the proper orientation required for the nucleophilic attack of ATP’s *γ*-phosphate (Johnson and Chen, 2018). Consistent with this hypothesis, introduction of a D793E mutation rescued ATPase activity at the degenerate site (Payen et al., 2003).

As expected, the binding site of bABCC1 undergoes significant changes during the transition from the inward- to outward-facing states. The ATP-bound, outward-facing conformation features a collapsed binding site caused by the LTC_4_-interacting residues pulling apart from each other (Johnson and Chen, 2018). The side chains of three residues of the P-pocket, R1196, N1244, and R1248, move away from LTC_4_’s glutathione moiety. R1248 forms a cation-*π* interaction with W1245, a key H-pocket residue in the inward-facing state. K332, H335, and Y440 from the P-pocket are collapsed into the LTC_4_ binding site while F594 flips toward the extracellular matrix. The changes in these four residues would likely push the LTC_4_ molecule toward the extracellular opening of the translocation pathway. Additional changes in the H-pocket help decrease affinity for LTC_4_ while opening the translocation pathway toward the extracellular matrix. The “tryptophan sandwich” between W553 and W1245 is abolished as the two residues are pulled away from each other. Y1242, positioned above LTC_4_’s lipid moiety in the inward-facing conformation, moves away from the binding site, opening the translocation pathway to the extracellular matrix. Although twice the concentration of LTC_4_ was used to prepare cryo-EM grids for this ATP-bound, outward-facing conformation as was used for the ATP-free, inward-facing conformation, no density was observed in the cryo-EM map that corresponded to LTC_4_, suggesting that in this ATP-bound, outward-facing conformation, LTC_4_ is released from bABCC1 before ATP hydrolysis occurs.

### The Catalytic Cycle of ABCC1

The most recent structure of ABCC1 showed bABCC1 under active turnover conditions (PDB: 6UY0). Wild-type bABCC1 was incubated with both LTC_4_ and ATP-Mg^2+^ prior to application to the cryo-EM grids (Wang et al., 2020). The resulting structure was essentially identical to the outward-facing E1454Q structure: in both structures, the binding site is pulled apart so that LTC_4_ can no longer bind and the intracellular gate is closed. The one important difference is that instead of two ATP molecules bound, the active turnover structure features an ATP molecule in the degenerate site and an ADP molecule in the consensus site. The nearly identical structures with ADP bound suggest that ATP hydrolysis is a fast step in the transport cycle, while the NBD separation happens more slowly, acting as the rate-limiting step of ABCC1’s catalytic cycle. ATP hydrolysis and release of inorganic phosphate do not stimulate ABCC1’s transition back to the inward-facing state.

Using single-molecule fluorescence spectroscopy, Wang et al. were able to further classify the catalytic cycle of bABCC1 (Wang et al., 2020). In the absence of ligand, ABCC1 may sample multiple inward-facing conformations and can also transition to the outward-facing state, albeit less frequently and for a shorter duration than was observed when nucleotides were present (Wang et al., 2020). ATP binding increased the rate of the inward-facing to outward-facing transition and stabilized the outward-facing conformation, which in turn decreased the reverse transition from outward to inward facing. Substrate increased the ATPase activity by accelerating the inward- to outward-facing transition, but substrate did not increase the rate of ATP hydrolysis or the outward- to inward-facing transition. Both the prehydrolytic state (two ATP molecules bound) and posthydrolytic state (one ATP, one ADP) shared the same high FRET value, suggesting that ATP hydrolysis is a quick step that is followed by a slow NBD dissociation back to the inward-facing state. This observation is consistent with other ABC transporters that exhibit a slow outward-facing to inward-facing transition. These observations, however, pertain to the bovine homolog, and differences likely exist between the bovine and human model.

### Structural Differences between ABCC1 and ABCB1 Reflect Differing Functions

Although ABCC1 and ABCB1 both contribute to MDR, there are significant differences in their function, some of which arise due to structural differences. ABCB1 transports hydrophobic substrates as well as a few weakly cationic compounds, while ABCC1 transports amphipathic, organic acids, and organic anions (Seelig et al., 2000; Johnson and Chen, 2017). ABCB1’s translocation pathway features hydrophobic residues with some acidic patches, whereas ABCC1’s translocation pathway is largely basic. ABCB1 and ABCC1 likely recruit substrates by different mechanisms as well. ABCB1 utilizes a “hydrophobic vacuum” mechanism, where substrates first partition into the cell membrane’s inner leaflet before entering ABCB1 (Higgins and Gottesman, 1992). Helices that span the translocation pathway of ABCB1 are flexible and are theorized to act as an intramembranous gate, allowing substrates to enter the transporter directly from the membrane (Jin et al., 2012; Kodan et al., 2014). The translocation of ABCC1 is only accessible from the cytoplasm as it is completely shielded from the membrane (Johnson and Chen, 2017, 2018). TM helices of ABCC1 are well ordered and contain no helical breaks. ABCC1 likely recruits substrates directly from the cytoplasm, likely due to the bipartite nature and plasticity of ABCC1’s single binding site.

### ABCC1 and Disease

*Abcc1*^−/−^ knockout mice have been used to interrogate ABCC1’s physiologic role as well as its role in drug response. *Abcc1*^−/−^ mice exhibited decreased inflammatory response, consistent with impaired LTC_4_ export, supporting ABCC1’s role in inflammatory and immunologic disease (Wijnholds et al., 1997; Cole, 2014a). *Abcc1*^−/−^ mice also exhibit increased levels of GSH in most tissues and are more sensitive to xenobiotics (Li et al., 2019). ABCC1 has been implicated as a protectant against methotrexate’s toxicity in the intestine after an ABCC1^−/−^ mouse model of colitis found that the disease was associated with higher mortality and severe epithelia damage (ten Hove et al., 2002; Kato et al., 2009; Cole, 2014b).

Single nucleotide polymorphisms (SNPs) identified for ABCC1 are mostly located in noncoding and intronic sequences (Perdu and Germain, 2001; Conrad et al., 2001; S. Ito et al., 2001; Saito et al., 2002; Oselin et al., 2003; Conseil et al., 2005). Additionally, none of these SNPs completely inactivate the transporter or prevent its expression at the plasma membrane. A polymorphism encoding the R433S mutation, located at the interface of the cytosol and TM8, reduced transport of organic anions; however, this mutation also paradoxically increased doxorubicin resistance (Conrad et al., 2001, 2002; Conseil et al., 2005). The C43S mutation, located in TM1 of TMD_0_, prevented ABCC1 from localizing to the plasma membrane (S. Ito et al., 2001; Leslie, Létourneau, et al., 2003; Conseil et al., 2005). HeLa cells expressing this mutation exhibited lower resistance to doxorubicin and sodium arsenite but showed no significant difference compared with wild-type for the transport of GSH, LTC_4_, E_2_17*β*G, and organic ions (Leslie, Létourneau, et al., 2003; Conseil et al., 2005).

The strongest association of ABCC1 with an unfavorable clinical outcome has been in neuroblastoma, the most common childhood extracranial solid tumor (Haber et al., 2006; Pajic et al., 2011; Cole, 2014a). ABCC1 has been implicated in human pathologies aside from cancer, including age-related macular degeneration, cardiovascular disease, some neurologic disorders, and regulation of oxidative stress (Cole, 2014a; Li et al., 2019). Recent genomic studies have identified certain ABCC1 polymorphisms associated with a greater/lesser severity of chronic obstructive pulmonary disease (COPD) (Siedlinski et al., 2009; Budulac et al., 2010). Five ABCC1 SNPs were identified from a COPD patient cohort; however, none of the identified SNPs translated to mutations within the protein (Siedlinski et al., 2009). An additional study found that ABCC1 had lower expression levels in bronchial epithelium of COPD patients than in healthy controls; however, no discussion of SNP’s effect on protein sequence or structure were included (Budulac et al., 2010). Two ABCC1 variants were identified in patients with anthracycline-induced cardiotoxicity, but one of these SNPs corresponded to a synonymous mutation while the second was annotated as occurring in the 3′-UTR (Semsei et al., 2012). Without data to clearly link (dys)function back to the protein structure or expression level, it is difficult to theorize how these reported SNPs affect ABCC1 in these diseases.

### ABCG2, the Breast Cancer Resistance Protein

The second member of subfamily G, ABCG2, was originally cloned from a doxorubicin-resistant breast cancer cell line where resistance independent of ABCB1 was strategically sought out by selecting for drug resistance in the presence of a ABCB1 inhibitor (Doyle et al., 1998). ABCG2 was initially named the breast cancer resistance protein, but three laboratories, including the aforementioned, vied to clone ABCG2 from the placenta as well as a mitoxantrone-resistant colon carcinoma cell line. Additional ABCG2 names were the placenta-specific ATP-binding cassette transporter (Allikmets et al., 1998) and mitoxantrone resistance protein (Miyake et al., 1999).

Possibly broader than ABCB1’s specificity, ABCG2 substrates include both hydrophobic and hydrophilic compounds, anions, and cations. ABCG2 is expressed on the apical membranes of various tissues and functions extrude xeno- and endobiotics including uric acid and porphyrins. While ABCG2 is expressed in the placenta, brain, intestine, liver, and kidney, it is also an important stem cell marker that was first functionally defined by Mullighan and Goodell (Goodell et al., 1996) but then molecularly identified and defined through overexpression and gene knockout studies by the laboratories of Sorrentino and Schuetz (Zhou et al., 2001, 2002). ABCG2 protects hematopoietic stem cells from xenobiotics to help maintain the stem cell pool. ABCG2 is also regulated by hypoxia, which is likely related to its protection of stem cells (Krishnamurthy et al., 2004). In 2008, genome-wide association studies determined that the C421A polymorphism (encoding Q141K, which has reduced expression and function) is associated with high uric acid levels, a basis for gout, suggesting another physiologically important role of ABCG2 as a urate transporter in humans (Dehghan et al., 2008). Elevated porphyrins, especially dietary chlorophyll degradation products, were an early phenotype of the ABCG2-deficient mouse (Jonker et al., 2002). A mouse model of erythropoietic protoporphyria when intercrossed with the ABCG2 deficient mouse showed reduced protoporphyrin IX levels in the skin that protected against erythropoietic protoporphyria-associated toxicity. This result affirmed ABCG2’s role as a protoporphyrin IX exporter (Wang et al., 2019). Due to its high expression in the placental syncytiotrophoblasts, ABCG2 likely provides a protective barrier for the fetus by preventing fetal exposure to xenobiotics (Fetsch et al., 2006).

Importantly, ABCG2 and ABCB1 seem to work in tandem at the blood-brain barrier to limit distribution of drugs to the brain (de Vries et al., 2007). ABCG2 can export anticancer drugs from cancer cells, and its high expression in human group 3 medulloblastoma cells was shown to cause chemoresistance (Morfouace et al., 2015). ABCG2 expression also correlates with a poor prognosis of acute myeloid leukemia (Damiani et al., 2006; Fukuda et al., 2017).

### ABCG2 Structures

Since 2017, several ABCG2 structures have been reported in different conformations [apo-inward-facing state, inhibitor/substrate-bound inward-facing state, substrate-bound turnover-1 state, substrate-bound turnover-2 (semiclosed) state, and outward-facing state] (Taylor et al., 2017; Jackson et al., 2018; Manolaridis et al., 2018; Orlando and Liao, 2020; Kowal et al., 2021; Yu et al., 2021; Rasouli et al., 2023). The structures in the apo-inward-facing state were captured by complexing the extracellular loop with Fab fragments from the monoclonal antibody 5D3 (PDB: 5NJ3 and 5NJG; Taylor et al., 2017). The apo-inward-facing structures reveal that TM helices are open and that the large binding cavity (cavity 1) is accessible from both the membrane and the cytosolic side. The extracellular cavity (cavity 2) is separated from the large substrate binding pocket by a “plug,” L554 and L555 residues. This plug is highly conserved among ABCG family members. These hydrophobic residues function like a lid, preventing substrate access to the extracellular cavity in the inward-facing state (Khunweeraphong et al., 2019). Recent studies without Fab fragments captured turnover states with both ATP and substrate in inward-facing like conformations (PDB: 7OJ8, 7OJI, 7OJH, 8BHT, and 8BI0; Yu et al., 2021; Rasouli et al., 2023). The turnover-1 structure has a substrate binding cavity accessible from the inner leaflet of the membrane. The turnover-2 structure reveals an almost fully occluded substrate binding cavity and semiclosed NBDs. Structural changes from inward-facing to turnover-2 conformation with substrate estrone 3-sulfate revealed how ATP binding affects the position of the interacting residues of the TMDs and substrate (PDB: 6HCO and 7OJ8). The ATP-bound structure in the outward-facing state was captured via mutation of the catalytic glutamate to glutamine (E211Q), showing a closed NBD dimer, a collapsed substrate binding cavity, and an opened extracellular cavity to the outside (PDB: 6HBU and 6HZM; Manolaridis et al., 2018). This ATP-bound structure supports the prevailing ATP-driven peristaltic extrusion model that dimerization of the NBDs by ATP binding promotes a transition to the outward-facing state where the substrate moves from the substrate binding cavity toward the extracellular cavity (McDevitt et al., 2008). Since the conformation observed in the ATP-bound ABCG2-E211Q mutant was not observed from ABCG2-WT turnover samples in the presence of substrate and ATP, this conformation in the ABCG2-E211Q mutant may be transient in a natural environment (Yu et al., 2021).

### The Binding Pocket of ABCG2

**Fig. 6.**
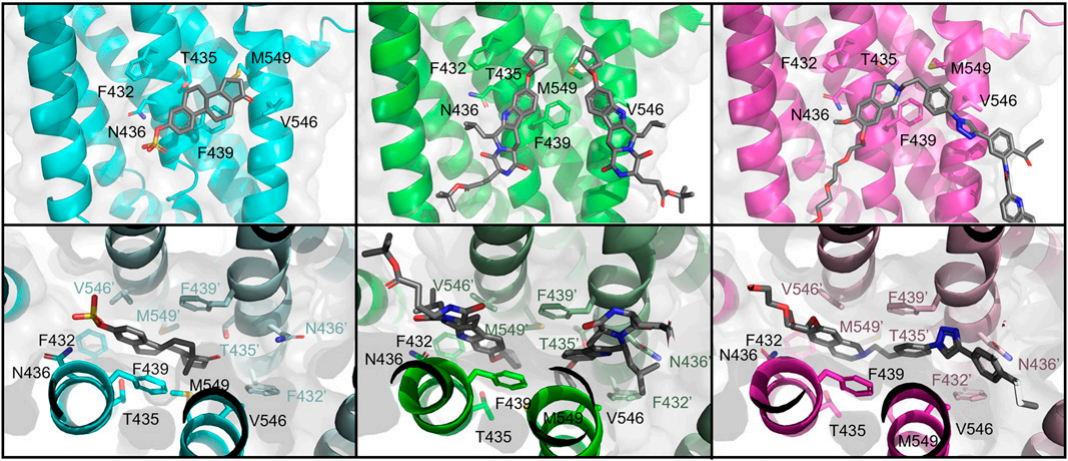
Structures of ligand-bound ABCG2. Top panels show cut-away from the binding interface while bottom panels show the view up the binding cavity. Ligands are shown as gray sticks. One monomer is shown in a bright color (TMD) while the second is shown in a pastel shade (TMD’). Left panels: Estrone 3-sulfate-bound ABCG2 (cyan, PDB: 6HCO). Middle panels: MZ29-bound ABCG2 (green, PDB: 6ETI). Right panels: MB136-bound ABCG2 (magenta, PDB: 6FEQ).

The substrate binding cavity of ABCG2 is formed by TM2 and TM5 (Manolaridis et al., 2018). ABCG2’s binding cavity, unlike ABCB1, is slit-like but hydrophobic and faces the cytoplasm. This structure appears optimal to accommodate flat, polycyclic, hydrophobic small molecules (Taylor et al., 2017). Available structures of ABCG2 demonstrate that the binding cavity accommodates one substrate molecule and one or two molecules of an inhibitor (Jackson et al., 2018; Manolaridis et al., 2018; Orlando and Liao, 2020; Kowal et al., 2021; Yu et al., 2021; Rasouli et al., 2023). ABCG2 inhibitors (e.g., MZ29; derived from well-known Ko143 and MB136; a derivative of tariquidar) and substrate (estrone 3-sulfate) enter the same binding pocket and have overlapping binding contacts (Fig. 6). This difference may account for why many ABCG2 ligands (e.g., tyrosine kinase inhibitors and tariquidar) possess both substrate and inhibitory characteristics, acting as ABCG2 substrates at low concentrations and competitively inhibiting the transport of some substrates at high concentrations. The ABCG2 inhibitors (MZ29 and MB136) and substrate (estrone 3-sulfate) form common hydrophobic interactions with residues F432, F439, V546, and M549 and polar or electrostatic interactions with T435 and N436 (Fig. 6). Recent studies identified residue F439 as indispensable for substrate transport and transport inhibition (Manolaridis et al., 2018; Gose et al., 2020). ABCG2 substrates and inhibitors form stacking interactions between two phenylalanine side chains, F439-F439’ (Gose et al., 2020).

Inhibitors seem to occupy a larger space in the binding cavity compared with the substrates. Small substrates like estrone 3-sulfate bind deep inside the binding cavity, below the leucine plug of ABCG2 (Manolaridis et al., 2018). Large inhibitors, like MB136, fully occupy the binding cavity, allowing for only a single molecule. Small inhibitors like MZ29 bind in pairs to fully occupy the binding pocket (Jackson et al., 2018). Compared with substrate, inhibitors make additional contacts involving residues of TM1, TM2, and TM5, which likely supports their higher affinity (Manolaridis et al., 2018).

### ABCB1, ABCG2, and ABCC1 Substrate Specificity

The cryo-EM structures of ABCG2 show an NBD and a TMD, and reveal tightly bound lipids interacting with the hydrophobic surface of the TMD (Taylor et al., 2017; Jackson et al., 2018). The transport activity of ABCG2 is modulated by the lipid environment and requires cholesterol for maximal activity, but, unlike its relative, ABCG1, it is not directly involved in cholesterol transport (Telbisz et al., 2013; Hegedüs et al., 2015; Jackson et al., 2018; Rasouli et al., 2023). ABCG2 has a relatively large internal cavity that is consistent with ABCG2’s ability to bind multiple ligand molecules (Taylor et al., 2017).

**Fig. 7.**
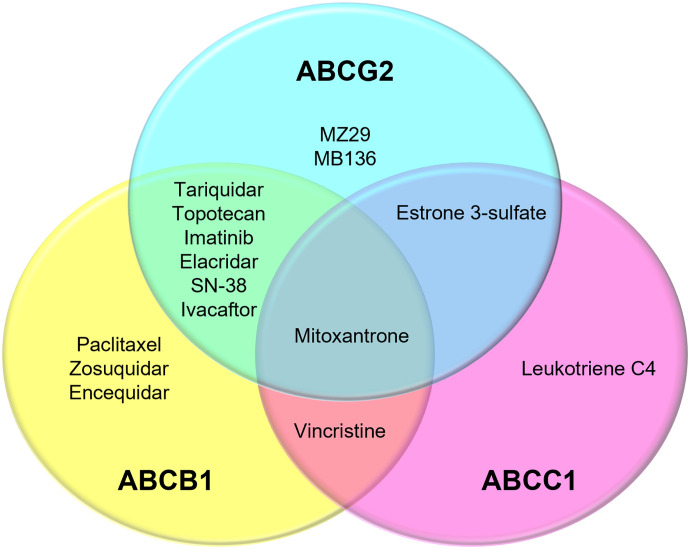
Venn diagram of selected compounds reported to bind ABCB1, ABCC1, and/or ABCG2 to show the overlapped specificity for the ABC transporters (based on reported structures).

Like ABCB1 and ABCC1, ABGG2 transports a variety of substrates, with some shared with ABCB1 and ABCC1 (Fig. 7, Table 1). Unlike ABCG2’s slit-like binding cavity, ABCB1 has a globular cavity, where large substrates such as paclitaxel (taxol) can be accommodated (Alam et al., 2019) (Fig. 3B). ABCC1 has a bipartite cavity, with the H-pocket accommodating hydrophobic substrates and the positively charged P-pocket accommodating glutathione (Fig. 5). In addition to the differences in substrate binding cavities, there are differences in the main axis of NBD rotation that is related to conformational changes in the TMDs. The main axis of NBD rotation is oriented parallel to the membrane in type II exporters including ABCG2 and perpendicular in type I exporters including ABCB1 and ABCC1 (Stockner et al., 2020). Studies in live cells or semipermeabilized cells provided some insight into the roles of substrate and ATP on conformational changes. An ABCB1, substrate cannot cause the conformational change in the absence of ATP, and ATP binding alone is insufficient for the conformational changes from the inward-facing to the outward-facing state (Futamata et al., 2020). In ABCG2, 5D3 reactivity, confocal microscopy-, and fluorescence correlation spectroscopy-based assays have revealed that the conformational changes of ABCG2 are induced by nucleotide binding and the transition is accelerated by ABCG2 substrate (Gyöngy et al., 2023). However, the relationship between structures obtained from nonphysiologic environments and protein function in the natural environment is not well established.

**TABLE 1:** Nonexhaustive list of pharmaceutical substrates and inhibitors of ABCB1 and ABCG2 Inhibitors were selected based on use in clinical trials. Substrates and inhibitors selected from Mo and Zhang, 2012; Ricci et al., 2015; Sajid et al., 2022; and Sissung et al., 2010.

ABCB1		ABCG2	
Substrates	Inhibitors	Substrates	Inhibitors
Antibiotics	CBT-1	Antibiotics	Tyrosine kinase inhibitors
Chloramphenicol	Dofequidar	Ciprofloxacin	Lapatinib
Erythromycin	Elacridar		Erlotinib
	Encequidar	Antimetabolites	
Cardiac glycosides	Laniquidar	Methotrexate	Other
Digoxin	Tariquidar		Elacridar
	Tesmilifene	Fluorescent dyes	
HIV protease inhibitors	Valspodar Zosuquidar	Hoechst 33342	
Ritonavir			
Saquinavir		HMG-CoA reductase inhibitors	
		Rosuvastatin	
Steroids			
Cortisol		Immunosuppressants	
Aldosterone		Sulfasalazine	
Dexamethasone			
		Natural compounds and toxins	
Taxanes		2-amino-1-methyl-6-phenylimidazo[4,5-b]pyridine	
Paclitaxel		Protoporphyrin IX	
Taxol		Uric acid	
			
Topoisomerase I and II inhibitors		Photosensitizers	
Anthracenes		Pheophorbide a	
Mitoxantrone			
Anthracyclines		Sulfate and glucuronide conjugates of xenobiotics	
Doxorubicin		Estradiol-17-beta-glucuronide	
Camptothecin analogs		Estrone 3-sulfate	
SN-38			
Topotecan		Topoisomerase I and II inhibitors	
Epipodophyllotoxins		Anthracenes	
Etoposide		Mitoxantrone	
		Anthracyclines	
Tyrosine kinase inhibitors		Doxorubicin	
Imatinib		Camptothecin analogs	
		SN-38	
Vinca alkaloids		Topotecan	
Vinblastine			
Vincristine		Tyrosine kinase inhibitors	
		Gefitinib	
		Imatinib mesylate	

### Nucleotide-Binding Domain and the Large Extracellular Loop 3 in ABCG2

The NBDs contain highly conserved motifs required for ATP binding and hydrolysis (Locher, 2016). The ATP-bound ABCG2-E211Q structure shows that the NBDs bind two ATP molecules and coordinate magnesium ions at their dimer interface (Manolaridis et al., 2018). The *γ*-phosphate of ATP interacts with three conserved side chains in the NBD, Q211 (catalytic glutamate in Walker B), H243 (Histidine switch), and Q126 (Q-loop). The magnesium ion also interacts with Q211 in Walker B (Manolaridis et al., 2018).

**Fig. 8.**
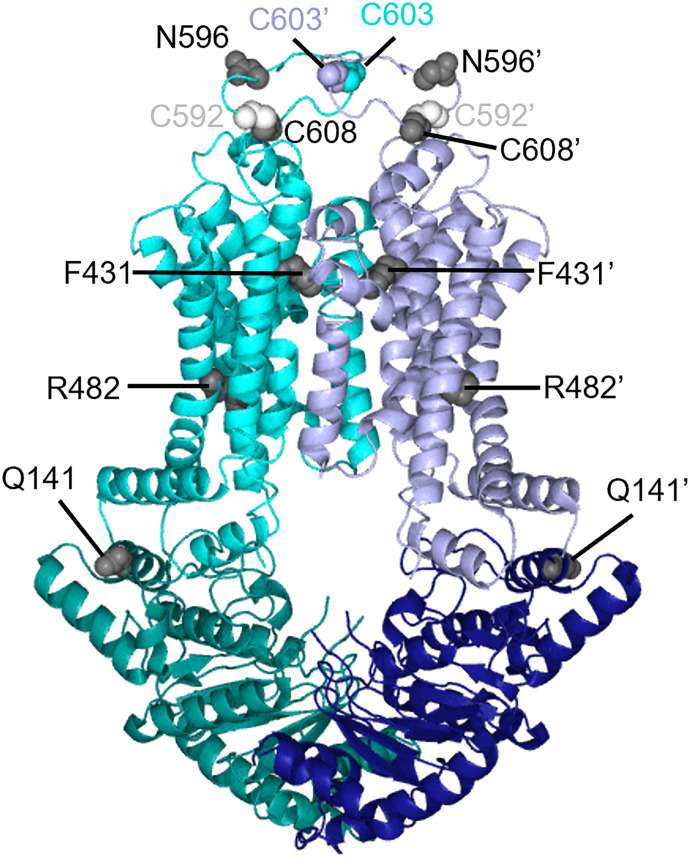
Residues of ABCG2 mapped onto the structure of ABCG2 (PDB: 6VXF) as gray spheres. C592 residue is shown as a white sphere to avoid confusion with C608.

In ABCG2, the cysteine residue C603 is located in the large extracellular loop 3 (ECL3) between TM5 and TM6 and forms an intermolecular disulfide bond (Fig. 8). The cysteine residues, C592 and C608, in the ECL3 form intramolecular disulfide bonds. N596 in ECL3 is modified by N-acetylglucosamine. Mutation of C603 disrupts intermolecular disulfide bond formation but does not affect membrane targeting or transport, suggesting the intermolecular disulfide bond of C603-C603’ is not essential for function (Henriksen et al., 2005). Orlando and Liao generated the copper phenanthroline-induced disulfide cross-linking assay to monitor the conformation changes of ABCG2 (Orlando and Liao, 2020). Conformationally sensitive residues in TM5 (V534 or A537) were mutated to cysteine. These residues were separately incorporated into a C603S ABCG2 background (Orlando and Liao, 2020). Treating these cysteine mutations with copper phenanthroline allowed the effect of substrate or inhibitor binding on conformation to be assessed. The mutation of either C592 or C608 abolished the intramolecular disulfide bond, reducing ABCG2 surface expression due to misfolding (Henriksen et al., 2005). The mutation of N596, which prevents glycosylation, enhanced ubiquitin-mediated proteasomal degradation, suggesting the essential role in ABCG2’s maturation (Nakagawa et al., 2009).

### Understanding the Effects of ABCG2 Mutations Based on Its Structure

The ABCG2 cryo-EM structures, complemented by molecular dynamics simulations, provide a powerful approach to understanding the dynamic effects of mutations on ABCG2 function. ABCG2 was initially cloned from a drug-resistant cancer cell (Doyle et al., 1998) but ultimately was found to have a different substrate profile than the ABCG2 protein cloned from the normal tissue (Allikmets et al., 1998). The ABCG2 from the drug-resistant cancer cells harbored a mutation at R482 (R482G/R482T) causing a gain of function (Honjo et al., 2001). Recent ABCG2 cryo-EM structures of the turnover state provided insight into this gain of function mutation by showing a structural role for R482 (Yu et al., 2021). R482 is located in TM3 and does not directly interact with bound substrate (Fig. 8). However, in the transition from the turnover-1 to the turnover-2 state, the R482 side chain contacts TM2, which contains F439, the key residue that interacts with both substrates and inhibitors in the binding cavity. This finding suggests that the altered substrate specificity of the R482 mutation might be allosteric (Yu et al., 2021). One of the most common polymorphisms, Q141K (rs2231142 C421A), is a risk factor for gout/hyperuricemia that also affects statin-based hyperlipidemia treatment (Chen et al., 2019). The Q141K mutation reduces ABCG2 surface expression due to folding and trafficking issues and shows loss of function (Mózner et al., 2019). In addition, ABCG2 cryo-EM structures show that the Q141 in the NBD faces the positively charged side of the amphipathic TM1a *α*-helix, and the positively charged Q141K side chain might cause electrostatic repulsion, thereby distorting TM1a and causing misfolding (Taylor et al., 2017). Another ABCG2 mutation, F431L (rs750568956 T1291C), is well expressed at the cell surface but has reduced transport function and inhibitor susceptibility (Yoshioka et al., 2007; Kawahara et al., 2010). An interpretation of this functional impairment, consistent with structural data, shows that F431 in TM2 is part of the substrate-binding pocket and directly interacts with the ABCG2 inhibitor MZ29, suggesting a role in ligand recognition (Jackson et al., 2018).

### Solute Carrier Transporter Superfamily

SLCs, the second largest family of membrane proteins are responsible for the movement of ions and small hydrophilic compounds across membranes (Wright and Lee, 2022). There are 446 different SLCs in humans subclassified into 70 families. Like ABC transporters, SLCs are integral membrane proteins. Unlike ABC transporters, SLCs act as secondary active transporters or facilitative transporters. It is estimated that 3% of approved drugs might target human SLCs, showing the importance of their structures for future drug development (Wright and Lee, 2022). Although ABC transporters are most famous for their pharmacological role in chemotherapeutics, the SLCs SLC19A1 and SLC29A1 also interact with these types of drugs.

## SLC19A1, the Reduced Folate Transporter

### Physiologic Importance of Folates and Their Transport

Reduced folates (vitamin B9 is a coenzyme) are essential for biosynthetic processes in one-carbon transfer reactions such as DNA/RNA synthesis and cellular methylation reactions (Crider et al., 2012; Bailey et al., 2015). Mammals lack a de novo folate synthesis pathway and rely on dietary folate from dark green leafy vegetables, liver, and folic-acid-fortified flour. Folates are essential for cell growth, proliferation, and differentiation, and their deficiency leads to developmental and neurologic disorders.

Folates rely on three carriers for uptake in mammals: reduced folate carrier (RFC/SLC19A1), proton-coupled folate transporter (SLC46A1), and the folate receptor (FR), the latter being a high affinity but low-capacity uptake route that probably is important in unique circumstances. SLC46A1 is highly expressed in the apical plasma membrane of the gastrointestinal tract and is responsible for dietary folate absorption but was initially inadvertently identified as a mammalian heme importer (Shayeghi et al., 2005) before being reported in cell as the proton-coupled folate transporter. Transport of folate mediated by SLC46A1 is proton-coupled, functioning best at acidic pH, unlike RFC (Zhao and Goldman, 2013; Matherly et al., 2014). Folate uptake by the FR occurs by endocytosis. Expression of FRs is limited in healthy tissues but is important for embryonic development (Zhao et al., 2009). Unlike SLC46A1 and FRs, whose role in folate transport seems tissue specific, SLC19A1 is ubiquitously expressed in tissues (e.g., brain, placenta, small intestine, colon, and kidney) (Zhao et al., 2009) and has a central role in folate uptake in various tissues. SLC19A1 acts as an antiporter, coupling extracellular folate uptake with the counter-transport of intracellular organic phosphate anions, including thiamine mono- and thiamine pyrophosphate (TPP), AMP, ADP, ATP, glucose 6-phosphate, and nicotinamide adenine dinucleotide (Goldman, 1971; Henderson and Zevely, 1983; Zhao, Gao, et al., 2001; Zhao et al., 2002).

### Identification and Role of SLC19A1

The reduced folate carrier (RFC/SLC19A1) was functionally characterized more than 50 years ago and exhibited temperature- and sodium-independent transport (Goldman et al., 1968; Goldman, 1969, 1971). The SLC19A1 cDNA was cloned by multiple laboratories between 1994 and 1995 (Dixon et al., 1994; Williams et al., 1994; Moscow et al., 1995; Prasad et al., 1995; Williams and Flintoff, 1995; Wong et al., 1995).

SLC19A1 is the main importer of reduced folates such as 5-methyltetrahydrofolate (5-MTHF), the reduced form of folate, the predominant natural folate in the diet and blood (Zhao et al., 2009). SLC19A1 shows a high affinity (micromolar) for 5-MTHF compared with folate (nearly millimolar). Proper fetal development requires SLC19A1 as SLC19A1 knockout is embryonic lethal (Zhao, Russell, et al., 2001; Hou and Matherly, 2014). Postnatal survival of the SLC19A1 knockout was mostly rescued by supplementing the mother’s diet with folate (Zhao, Russell, et al., 2001). This observation is consistent with SLC19A1’s location in the fetal-facing basal membrane of the placental syncytiotrophoblasts where it contributes to transplacental import of folate from the maternal blood to fetal circulation. SLC19A1 dysfunction has been associated with fetal abnormalities, megaloblastic anemia, neurologic disorders, cardiovascular disease, and cancer (Yang et al., 2003; Matherly and Hou, 2008; Svaton et al., 2020).

Folate is required for rapidly dividing cancer cells, and SLC19A1 has been exploited to mediate chemotherapeutic drug uptake (Matherly et al., 2007). SLC19A1 imports antifolate therapeutics such as methotrexate (MTX), PT523, pralatrexate, raltitrexed, and pemetrexed (PMX) (Matherly et al., 2007; Kanarek et al., 2018; O’Connor et al., 2021). SLC19A1’s genetic variants affect antifolate response, showing a crucial role in the treatment of cancers and inflammatory diseases such as rheumatoid arthritis, psoriasis, and inflammatory bowel disease (Zhao and Goldman, 2003; Jansen et al., 2004; Matherly et al., 2007; Yee et al., 2010; Kanarek et al., 2018; O’Connor et al., 2021). The use of antifolates during pregnancy is contraindicated because of the developmental risk to the fetus. Understanding how antifolates engage SLC19A1 may promote the rational design of therapeutics that selectively target SLC19A1.

Recently, SLC19A1 acquired a new endogenous function when it was identified as an importer of immunomodulatory cyclic dinucleotides (CDNs) by an unbiased, genome-wide CRISPR screen (Luteijn et al., 2019; Ritchie et al., 2019). Endogenous CDN (2’, 3’-cGAMP), produced through cyclic GMP-AMP synthase, as well as bacterial and synthetic CDN analogs bind and activate the stimulator of interferon genes (STING) pathway (Luteijn et al., 2019; Ritchie et al., 2019). The immune-system sensor protein, STING, located in the endoplasmic reticulum, has immunomodulatory functions. However, how the charged extracellular CDNs cross the plasma membrane to activate STING remains unclear. The identification of SLC19A1 as an importer of CDNs has implications for cancer immunotherapy and host-pathogen response (Luteijn et al., 2019; Ritchie et al., 2019). CDN uptake is inhibited by folates and antifolate, suggesting that they compete for a binding site on SLC19A1 (Luteijn et al., 2019).

### SLC19A1 Structures

The structural basis of substrate recognition by SLC19A1 was unknown until recently. Multiple laboratories have reported the cryo-EM structures of human SLC19A1 in substrate-free and apo-states (PDB: 8DEP, 7XPZ, and 8HII, Dang et al., 2022; Wright et al., 2022; Zhang et al., 2022) but also in complexes with the natural folate (5-MTHF) (PDB: 8GOE and 8HIJ; Dang et al., 2022; Zhang et al., 2022), antifolates PMX and MTX (PDB: 8GOF and 7TX6; Wright et al., 2022; Zhang et al., 2022), and CDNs (PDB: 7XQ0, 7XQ1, and 7XQ2; Zhang et al., 2022), and TPP (PDB: 8HIK; Dang et al., 2022), thereby providing a basis for understanding of SLC19A1-mediated substrate recognition and transport. Like the substrate-free apo-structure of SLC19A1, all SLC19A1-substrate complexes adopt an inward-facing conformation. Through molecular dynamics simulations-guided mutagenesis and functional studies, key substrate binding residues for SLC19A1 were determined. SLC19A1 exhibits the canonical major facilitator superfamily (MFS) fold, which is comprised of 12 transmembrane TM segments separated into the N-terminal (TM1–TM6) and C-terminal (TM7–TM12) domains (Wright et al., 2022). According to the alternative access “rocker-switch” mechanism (Drew et al., 2021) (Fig. 2), SLC19A1 toggles between an inward-facing and outward-facing conformation to both import and export substrates in a counterexchange fashion (e.g., exchanging extracellular 5-MTHF with intracellular organic anions). SLC19A1 has a deep polar substrate-binding cavity that extends across the membrane with positively charged amino acids at the cytosolic entrance and interior cavity (Dang et al., 2022; Wright et al., 2022; Zhang et al., 2022). The location of these charged residues is consistent with the function of this organic anion antiporter. The residues of the large intracellular loop connecting TM6 and TM7 were poorly resolved in SLC19A1’s cryo-EM structure, likely reflecting the high mobility of this loop. This intracellular loop is tightly embedded in a surface groove and might form extensive polar and hydrophobic interactions. This loop has also been proposed to ensure appropriate spacing between the N-terminal and C-terminal domains for optimal transporter function (Matherly et al., 2014, Zhang et al., 2022).

### Substrate Recognition of SLC19A1

Single molecules of folate (5-MTHF), antifolates (MTX and PMX), and TPP bind in an upright conformation to the narrow, upper portion of SLC19A1 (Dang et al., 2022; Wright et al., 2022; Zhang et al., 2022). Surprisingly, CDNs bound SLC19A1 as a tightly packed dimer in the wider intracellular entrance of SLC19A1, extending to the middle of the transmembrane region (Zhang et al., 2022). Therefore, CDNs occupy a distinct binding site with minimal overlap with the monomeric folate/antifolate binding site: CDN binds the large polar cavity rather than the narrower folate/antifolate binding pocket. This knowledge does not account for why CDNs compete with folate/antifolate for transport. Further, it is unclear how SLC19A1 transports the CDNs across the membrane, but both CDN molecules form *π*-*π* stacking, hydrogen bonding, and charge interactions with residues from N- and C-terminal domains of SLC19A1, supporting a unique dual-molecular recognition mechanism (Zhang et al., 2022).

**Fig. 9.**
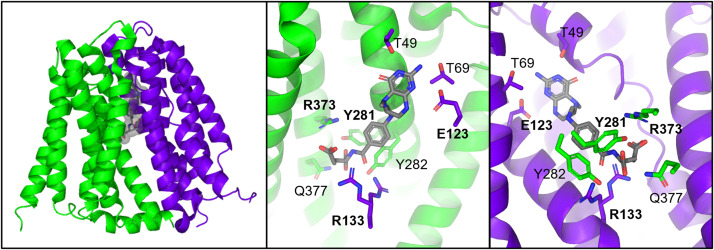
Left panel: Structure of 5-MTHF-bound SLC19A1 (PDB: 8GOE). N-terminal domain is shown in green, C-terminal domain in purple. Residues important for binding are shown in bold. Middle panel: Cutaway of ligand binding site to remove C-terminal domain. Right panel: Cutaway of ligand binding site to remove N-terminal domain.

The TM1, TM4, TM7, and TM10 of SLC19A1 form the 5-MTHF binding site (Dang et al., 2022; Zhang et al., 2022). To determine the importance of binding site residues with 5-MTHF, mutants of the interacting residues were assessed for MTX uptake activity. Substitutions of E123, R133, Y281, and R373 with alanine abolished the MTX uptake activity of SLC19A1 (Dang et al., 2022; Wright et al., 2022). The extracellular substrate, 5-MTHF, and intracellular substrate, TPP, bound to the identical site with residues E123, R133, Y281, and R373 forming polar or electrostatic interactions with 5-MTHF (PDB: 8GOE and 8HIJ) (Fig. 9) and TPP (PDB:8HIK) (Dang et al., 2022; Zhang et al., 2022). This result suggested that these residues are crucial for substrate recognition. E123, R133, Y281, and R373 are all highly conserved in SLC19A1 among different species (Dang et al., 2022). Structural insights from SLC19A1 can aid in the design of more effective therapeutics and may shed light on the mechanism of disease-related mutations. However, to fully understand SLC19A1 transport mechanism, alternative structural conformations are needed.

## SLC29A1, an Equilibrative Nucleoside Transporter

The equilibrative nucleoside transporters are transporters belonging to the solute carrier transporter family SLC29 (Baldwin et al., 2004; Young et al., 2013; Pastor-Anglada et al., 2022). As their name implies, equilibrative nucleoside transporters are major players in the transport of nucleobases (adenine) and nucleosides (adenosine) through a rocker-switch mechanism (Fig. 2), whereby the two halves of the transporter undergo rigid body rearrangements to alternate solvent access of the transporter cavity to either side of the lipid bilayer (Wright and Lee, 2019, 2021, 2022; Qureshi et al., 2020; Drew et al., 2021). In humans, the SLC29 family proteins are responsible for transporting several Food and Drug Administration- or European Medicines Agency-approved antihypertensive, antiviral, and anticancer drugs (Baldwin et al., 2004; Young et al., 2013; Wright and Lee, 2019; Pastor-Anglada et al., 2022) Broadly, SLC29A1 is an energy-independent facilitative transporter responsible for transport of purine and pyrimidine nucleosides. SLC29A1 is not a high-affinity transporter and exhibits K_M_ values ranging from 50 to 480 *μ*M for nucleosides (e.g., adenosine, guanosine, inosine, uridine, cytidine, thymidine) and > 10-fold lower affinity for nucleobases (e.g., hypoxanthine, adenine, guanine, uracil, and thymine).

### SLC29A1’s Role in Health and Disease

The Augustine blood type, characterized by SLC29A1 mutations, was established as a blood group in 2015, although the antigen was first identified in 1967 (Daniels et al., 2015; Daniels, 2022). SLC29A1-null individuals appear healthy, although pseudogout and ectopic calcification during aging have been reported (Daniels et al., 2015; Pastor-Anglada et al., 2022). SLC29A1 deficiency has not been listed in the OMIM catalog at present. SLC29A1 transports adenosine, a vital signaling molecule in human physiology, with roles in pain, inflammation, ischemia, epilepsy, and alcohol preference (Baldwin et al., 2004; Young et al., 2013; Wright and Lee, 2019). Given SLC29A1’s role in regulating adenosine levels inside and outside the cell, adenosine reuptake inhibitors have been used to block SLC29A1 function for vasodilation, antithrombosis, hypertension, and renal disorders (Yoshida et al., 1994; Young et al., 2008; FitzGerald 1987; Boswell-Casteel and Hays, 2017; Wright and Lee, 2019). Previous studies have reported a high correlation between SLC29A1 expression and survival outcome of pancreatic cancer patients receiving gemcitabine, a nucleoside analog (Marcé et al., 2006; Spratlin et al., 2004; Farrell et al., 2009; Maréchal et al., 2009; Hagmann et al., 2010; Tanaka et al., 2010; Voutsadakis, 2011; Greenhalf et al., 2014). Several protein kinase inhibitors such as imatinib, a Bcr-Abl tyrosine kinase inhibitor used to treat chronic myelogenous leukemia, have been shown to effectively inhibit SLC29A1 as well as SLC29A2, although the mechanism is undetermined and may be related to effects on post-translational modification (Woodahl et al., 2008). SLC29A1, along with SLC29A2, are inhibited by nitrobenzylthioinosine (NBMPR), as well as dipryridamole, a vasodilator and blood thinner; dilazep, a vasodilator used in cardiopathy and renal disorders; and draflazine, a vasodilator (Wright and Lee, 2019). Recently, the structure of SLC29A1 was published, allowing insight into how two of these compounds, NBMPR (PDB: 6OB6) and dilazep (PDB: 6OB7), bind and inhibit the transporter.

### SLC29A1 Structures

SLC29A1 contains 11 transmembrane helices, an intracellular N-terminus, and an extracellular C-terminus (Baldwin et al., 2004; Young et al., 2013; Wright and Lee, 2019). SLC29A1 is structured into two pseudo-symmetrical bundles where 6-TM helices compose the first bundle (N-domain), and the remaining 5 TMs compose the second bundle (C-domain). SLC29A1 contains a single N-linked glycosylation site; however, glycosylation is not required for proper protein function or trafficking to the plasma membrane (Young et al., 2013). SLC29A1’s folding is highly similar to the first 11 TM helices of the MFS transporters, a SLC subgroup with a 6 + 6 pseudo-symmetric topology (Wright and Lee, 2019). However, the absence of the final TM helix in SLC29A1 causes distinct deviations from the general MFS architecture.

**Fig. 10.**
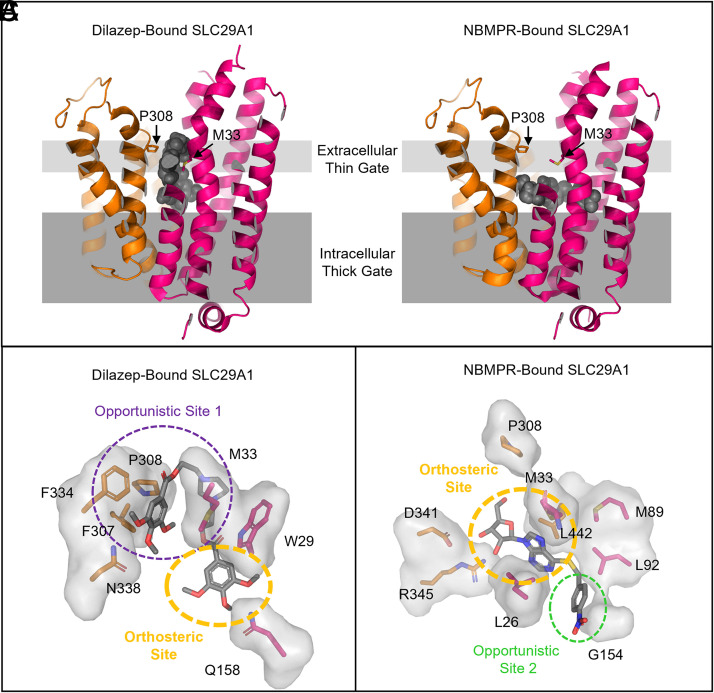
(A) Structure of SLC29A1 bound to Dilazep (PDB: 6OB7) and NBMPR (PDB: 6OB6). N-terminal domain is shown in orange, C-terminal domain is shown in hot pink, and ligands are shown as gray spheres. Dilazep occupies the orthosteric site and opportunistic site 1 (B) while NBMPR occupies the orthosteric site and opportunistic site 2 (C).

In the inhibitor-bound SLC29A1 structures, SLC29A1 exhibits the outward-facing conformations (Wright and Lee, 2019). The extracellular side of the transporter constricts into its narrowest point, termed the “extracellular thin gate,” between TM1’s M33 and TM7’s P308 (Fig. 10A). This gate blocks ligand from easily releasing into the extracellular side of the membrane. On the cytosolic side, TM4, TM5, TM10, and TM11 exhibit extensive hydrophobic contacts that fully prevent solvent access. Polar and charged interactions stabilize this cytosolic gate, termed the “intracellular thick gate.” R111 and E428 of the intracellular thick gate are evolutionarily conserved, suggesting an important role for these residues (Wright and Lee, 2019). However, these sites differ from the highly conserved “A motif” of MFS, suggesting a difference in mechanism between SLC29 transporters and MFS.

### Inhibition of SLC29A1

NBMPR and dilazep bind overlapping but different binding sites within SLC29A1 based on published crystal structures (Fig. 10A) (Wright and Lee, 2019). Dilazep adopts a crescent formation within the binding site (PDB: 6OB7), with one of dilazep’s trimethoxyphenyl rings located deep within the central cavity and the other close to the extracellular side. These positions are termed the “orthosteric site” and “opportunistic site 1,” respectively (Fig. 10B) (Wright and Lee, 2019). Dilazep makes contacts with W29 (TM1) and Q158 (TM4) in the orthosteric site, while *π*-*π* stacking between P307 (TM7), F334 (TM8), and dilazep’s trimethoxyphenyl ring contributes to binding within opportunistic site 1 (Fig. 10C). Hydrogen bonding between N338 (TM8) also occurs in this opportunistic site 1. Dilazep’s central diazepane ring forms hydrophobic contacts with M33 (TM1), previously found to determine isoform specificity for dilazep and dipyridamole (Visser et al., 2002; Visser, Baldwin, et al., 2005; Wright and Lee, 2019). In addition, mutation of W29, F334, and N338 have been previously shown to alter SLC29A1 transport of dilazep, which is supported by the structure of the binding pockets (Visser et al., 2007; Paproski et al., 2008; Wright and Lee, 2019).

NBMPR binds an overlapping but unique site in SLC29A1 (PDB:6OB6; Wright and Lee, 2019). The central cavity of SLC29A1 is occupied by the thioinosine moiety of NBMPR, while the 2’- and 3′-OH groups of NBMPR interact with R345 and D341, two highly conserved residues (Arastu-Kapur et al., 2003). N-1 and N-3 amino groups of the thioinosine moiety interact with Q158, another conserved residue proposed to play a role in nucleobase recognition (Wright and Lee, 2019). Several hydrophobic residues in TM1, TM2, and TM11 (L26, M89, L92, and L442) surround the purine moiety of NBMPR, with L26 and L442 forming a sandwich with the purine ring. Previously, the L442I mutation was reported to switch SLC29A1 preference to uridine, a pyrimidine, over adenosine, a purine, which is consistent with the reported structure (Paproski et al., 2008; Wright and Lee, 2019). M89 and L92 have also been found important for binding NBMPR and other purines (Endres et al., 2004; Endres and Unadkat, 2005). NBMPR occupies a second cavity that dilazep does not, termed “opportunistic site 2.” In this site, the *p-*nitrobenzyl ring of NBMPR sits directly next to G154. In SLC29A2 and SLC29A3, the equivalent residue to G154 is serine, which results in a ∼2500-fold decrease in NBMPR inhibition. Mutation of this residue in SLC29A1’s model narrows the hydrophobic cavity, preventing NBMPR’s *p-*nitrobenzyl ring from binding. As a result, it has been proposed that G154 plays a role in SLC29A1 specificity for NBMPR (SenGupta and Unadkat, 2004; Wright and Lee, 2019).

Structures of both the NBMPR- and dilazep-bound SLC29A1 suggest that opportunistic site 1 is used for dilazep binding only but not NBMPR binding (Wright and Lee, 2019). Mutation studies of M33 in the opportunistic site showed a threefold decrease in K_D_ for dilazep but no change in K_D_ for NBMPR. The F307A mutation resulted in a ∼90-fold decrease in affinity while the more conservative F307Y mutation resulted in a ∼4-fold decrease in affinity for dilazep. Conversely, neither F307A nor F307Y affected NBMPR binding in SLC29A1 (Visser et al., 2002; Visser, Zhang, et al., 2005). Mutations of Q158 in the orthosteric site to serine or asparagine showed both mutations inhibit binding of a radiolabeled NBMPR analog. These results suggested that mutation in the orthosteric site disrupted NBMPR binding, while mutation in the opportunistic site 1 disrupted dilazep binding but not NBMPR binding.

The overlapping but distinct binding sites of dilazep and NBMPR create two distinct inhibitory mechanisms of SLC29A1 (Wright and Lee, 2019). Both the dilazep and NBMPR structures exhibit an outward-facing conformation. To transition to the inward-facing state, the extracellular thin gate forms upon substrate binding and then C- and N-terminal domains rearrange to form the intracellular thick gate (Quistgaard et al., 2016). Dilazep occupies opportunistic site 1, which is located at the region including extracellular thin gate. Dilazep acts sterically to prevent the extracellular thin gate from forming, which prevents the transition from the outward facing to outward occluded, an intermediate in the transition from outward- to inward-facing conformation. This mechanism of action has been used previously in competitive inhibitors in sodium-coupled neurotransmitter transporters and MFS transporters (Krishnamurthy and Gouaux, 2012; Penmatsa et al., 2013; Deng et al., 2014; Coleman et al., 2016). Conversely, NBMPR does not occupy opportunistic site 1, and so NBMPR binding does not sterically hinder the extracellular thin gate from forming. However, the *p*-nitrobenzyl ring of NBMPR protrudes into opportunistic site 2, a deep hydrophobic cavity in the N-domain. Opportunistic site 2 is surrounded by TM1, TM3, and TM4, which are important for the conformational changes of the rocker-switch mechanism (Nomura et al., 2015; Quistgaard et al., 2016; Hirschi et al., 2017). NBMPR’s *p*-nitrobenzyl ring prevents the conformational rearrangement of these TM’s, blocking transition to the inward-facing state and transport across the membrane. NBMPR’s binding features a unique mechanism whereby a competitive inhibitor used an extended moiety for allosteric control of a conformational transition (Wright and Lee, 2019). These distinct binding modes of SLC29A1 allow two different options for pharmacological design of SLC29A1 inhibitors.

## Conclusions

In conclusion, drug transporters are crucial to our understanding of the pharmacokinetics and pharmacodynamics of drugs. Advances in our understanding of transporter structure and function are presently opening new avenues for drug development. The new discoveries of ligand-bound transporter transition states are likely to advance the development of more effective drugs to either use or evade transporters as well as provide insights into how transporter variation affects function. The improvement in both software and hardware has revolutionized membrane protein structural determination by cryo-EM, as well as more classic techniques of X-ray crystallography and NMR. These advances catalyzed the “resolution revolution,” thereby accelerating the membrane transporter structural determination and allowing for the determination of several of the structures previously discussed in this review. We now understand much more about how these transporters function and interact with their ligands compared with what was proposed by Jardetzky in 1966. However, as discussed previously, the structures of several pharmacologically interesting proteins are still inherently difficult to determine, even in the age of cryo-EM. Changes to the protein to aid in structure determination, such as stabilization with a Fab fragment, removal of flexible regions, or study of a better-behaved homolog, all come at a “cost” to the understanding of the physiologically relevant protein structure. Deep learning-based protein structure prediction algorithms, such as those developed by DeepMind’s AlphaFold 2, have the potential to predict the structure of membrane transporters, but because these are nonligand bound states, they are unlikely to provide the functional insights on their catalytically relevant intermediate states. Perhaps future versions of AlphaFold will include structural predictions based on the growing body of ligand-bound transporter structures. The most complete structure is likely a holistic view of multiple techniques. However, more detailed structural information will not change the fact that, in cells transporters do not function in isolation and often have overlapping substrates and compensatory systems in the cell that make them challenging drug targets, at least at present.

## Abbreviations

5-MTHF: 5-methyltetrahydrofolate
ABC: ATP binding cassette
AdoRI: adenosine reuptake inhibitor
bABCC1: bovine ABCC1
BCRP: breast cancer resistance protein
CDN: cyclic dinucleotide
COPD: chronic obstructive pulmonary disease
DEER: double electron-electron resonance
ECL3: extracellular loop 3
FR: folate receptor
GSH: glutathione
HES: high-energy state
LTC_4_: leukotriene C_4_
MDR: multidrug resistance
MDR1: multidrug resistance protein 1
MFS: major facilitator superfamily
MTX: methotrexate
NBD: nucleotide-binding domain
NBMPR: nitrobenzylthioinosine
PMX: pemetrexed
RFC: reduced folate carrier
SLC: solute carrier
SNP: single nucleotide polymorphism
STING: stimulator of interferon genes
TM: transmembrane helix
TMD: transmembrane domain
TPP: thiamine pyrophosphate

## References

[B1] Abdul-Ghani R Serra V Györffy B Jürchott K Solf A Dietel M Schäfer R (2006) The PI3K inhibitor LY294002 blocks drug export from resistant colon carcinoma cells overexpressing MRP1. Oncogene 25**:** 1743–1752. 16288223 10.1038/sj.onc.1209201

[B2] Alam A Kowal J Broude E Roninson I Locher KP (2019) Structural insight into substrate and inhibitor discrimination by human P-glycoprotein. Science (1979) 363**:** 753–756. 10.1126/science.aav7102 PMC6800160 30765569

[B3] Alam A Küng R Kowal J McLeod RA Tremp N Broude EV Roninson IB Stahlberg H Locher KP (2018) Structure of a zosuquidar and UIC2-bound human-mouse chimeric ABCB1. Proc Natl Acad Sci USA 115**:** E1973–E1982. 29440498 10.1073/pnas.1717044115 PMC5834697

[B4] Allikmets R Schriml LM Hutchinson A Romano-Spica V Dean M (1998) A human placenta-specific ATP-binding cassette gene (ABCP) on chromosome 4q22 that is involved in multidrug resistance. Cancer Res 58**:** 5337–5339. 9850061

[B5] Ambudkar SV Kim IW Xia D Sauna ZE (2006) The A-loop, a novel conserved aromatic acid subdomain upstream of the Walker A motif in ABC transporters, is critical for ATP binding. FEBS Lett 580**:** 1049–1055. 16412422 10.1016/j.febslet.2005.12.051

[B6] Arastu-Kapur S Ford E Ullman B Carter NS (2003) Functional analysis of an inosine-guanosine transporter from Leishmania donovani. The role of conserved residues, aspartate 389 and arginine 393. J Biol Chem 278**:** 33327–33333. 12807872 10.1074/jbc.M305141200

[B7] Bailey LB Stover PJ McNulty H Fenech MF Gregory JF 3rd Mills JL Pfeiffer CM Fazili Z Zhang M Ueland PM (2015) Biomarkers of nutrition for development-folate review. J Nutr 145**:** 1636S–1680S. 26451605 10.3945/jn.114.206599 PMC4478945

[B8] Bakos E Evers R Sinkó E Váradi A Borst P Sarkadi B (2000) Interactions of the human multidrug resistance proteins MRP1 and MRP2 with organic anions. Mol Pharmacol 57**:** 760–768. 10727523 10.1124/mol.57.4.760

[B9] Bakos E Evers R Szakács G Tusnády GE Welker E Szabó K de Haas M van Deemter L Borst P Váradi A (1998) Functional multidrug resistance protein (MRP1) lacking the N-terminal transmembrane domain. J Biol Chem 273**:** 32167–32175. 9822694 10.1074/jbc.273.48.32167

[B10] Baldwin SA Beal PR Yao SY King AE Cass CE Young JD (2004) The equilibrative nucleoside transporter family, SLC29. Pflugers Arch 447**:** 735–743. 12838422 10.1007/s00424-003-1103-2

[B11] Bickers SC Benlekbir S Rubinstein JL Kanelis V (2021) Structure of Ycf1p reveals the transmembrane domain TMD0 and the regulatory region of ABCC transporters. Proc Natl Acad Sci USA 118**:** e2025853118. 34021087 10.1073/pnas.2025853118 PMC8166025

[B12] Biedler JL Riehm H (1970) Cellular resistance to actinomycin D in Chinese hamster cells in vitro: cross-resistance, radioautographic, and cytogenetic studies. Cancer Res 30**:** 1174–1184. 5533992

[B13] Binkhathlan Z Lavasanifar A (2013) P-glycoprotein inhibition as a therapeutic approach for overcoming multidrug resistance in cancer: current status and future perspectives. Curr Cancer Drug Targets 13**:** 326–346. 23369096 10.2174/15680096113139990076

[B14] Boswell-Casteel RC Hays FA (2017) Equilibrative nucleoside transporters—a review. Nucleosides Nucleotides Nucleic Acids 36**:** 7–30. 27759477 10.1080/15257770.2016.1210805 PMC5728162

[B15] Brózik A Hegedüs C Erdei Z Hegedüs T Özvegy-Laczka C Szakács G Sarkadi B (2011) Tyrosine kinase inhibitors as modulators of ATP binding cassette multidrug transporters: substrates, chemosensitizers or inducers of acquired multidrug resistance? Expert Opin Drug Metab Toxicol 7**:** 623–642. 21410427 10.1517/17425255.2011.562892

[B16] Bruggemann EP Currier SJ Gottesman MM Pastan I (1992) Characterization of the azidopine and vinblastine binding site of P-glycoprotein. J Biol Chem 267**:** 21020–21026. 1356986

[B17] Budulac SE Postma DS Hiemstra PS Kunz LI Siedlinski M Smit HA Vonk JM Rutgers B Timens W Boezen HM; Groningen Leiden Universities Corticosteroids in Obstructive Lung Disease (GLUCOLD) Study Group (2010) Multidrug resistance-associated protein-1 (MRP1) genetic variants, MRP1 protein levels and severity of COPD. Respir Res 11**:** 60. 20487524 10.1186/1465-9921-11-60 PMC2882908

[B18] Cartwright TA Campos CR Cannon RE Miller DS (2013) Mrp1 is essential for sphingolipid signaling to P-glycoprotein in mouse blood-brain and blood-spinal cord barriers. J Cereb Blood Flow Metab 33**:** 381–388. 23168528 10.1038/jcbfm.2012.174 PMC3587808

[B19] Chang XB (2010) Molecular mechanism of ATP-dependent solute transport by multidrug resistance-associated protein 1. Methods Mol Biol 596**:** 223–249. 19949927 10.1007/978-1-60761-416-6_11

[B20] Chen L Manautou JE Rasmussen TP Zhong XB (2019) Development of precision medicine approaches based on inter-individual variability of BCRP/*ABCG2*. Acta Pharm Sin B 9**:** 659–674. 31384528 10.1016/j.apsb.2019.01.007 PMC6664102

[B21] Cole SPC (2014a) Multidrug resistance protein 1 (MRP1, ABCC1), a “multitasking” ATP-binding cassette (ABC) transporter. J Biol Chem 289**:** 30880–30888. 25281745 10.1074/jbc.R114.609248 PMC4223294

[B22] Cole SPC (2014b) Targeting multidrug resistance protein 1 (MRP1, ABCC1): past, present, and future. Annu Rev Pharmacol Toxicol 54**:** 95–117. 24050699 10.1146/annurev-pharmtox-011613-135959

[B23A] Cole SP Bhardwaj G Gerlach JH Mackie JE Grant CE Almquist KC Stewart AJ Kurz EU Duncan AM Deeley RG (1992) Overexpression of a transporter gene in a multidrug-resistant human lung cancer cell line. Science 258**:** 1650–1654. 1360704 10.1126/science.1360704

[B23] Cole SPC Deeley RG (2006) Transport of glutathione and glutathione conjugates by MRP1. Trends Pharmacol Sci 27**:** 438–446. 16820223 10.1016/j.tips.2006.06.008

[B24] Coleman JA Green EM Gouaux E (2016) X-ray structures and mechanism of the human serotonin transporter. Nature 532**:** 334–339. 27049939 10.1038/nature17629 PMC4898786

[B25] Conrad S Kauffmann HM Ito K Deeley RG Cole SPC Schrenk D (2001) Identification of human multidrug resistance protein 1 (MRP1) mutations and characterization of a G671V substitution. J Hum Genet 46**:** 656–663. 11721885 10.1007/s100380170017

[B26] Conrad S Kauffmann HM Ito K Leslie EM Deeley RG Schrenk D Cole SPC (2002) A naturally occurring mutation in MRP1 results in a selective decrease in organic anion transport and in increased doxorubicin resistance. Pharmacogenetics 12**:** 321–330. 12042670 10.1097/00008571-200206000-00008

[B27] Conseil G Deeley RG Cole SPC (2006) Functional importance of three basic residues clustered at the cytosolic interface of transmembrane helix 15 in the multidrug and organic anion transporter MRP1 (ABCC1). J Biol Chem 281**:** 43–50. 16230346 10.1074/jbc.M510143200

[B28] Conseil G Deeley RG Cole SPC (2005) Polymorphisms of MRP1 (ABCC1) and related ATP-dependent drug transporters. Pharmacogenet Genomics 15**:** 523–533. 16006996 10.1097/01.fpc.0000167333.38528.ec

[B29] Conseil G Rothnie AJ Deeley RG Cole SPC (2009) Multiple roles of charged amino acids in cytoplasmic loop 7 for expression and function of the multidrug and organic anion transporter MRP1 (ABCC1). Mol Pharmacol 75**:** 397–406. 19015228 10.1124/mol.108.052860

[B30] Cordon-Cardo C O’Brien JP Casals D Rittman-Grauer L Biedler JL Melamed MR Bertino JR (1989) Multidrug-resistance gene (P-glycoprotein) is expressed by endothelial cells at blood-brain barrier sites. Proc Natl Acad Sci USA 86**:** 695–698. 2563168 10.1073/pnas.86.2.695 PMC286540

[B31] Crider KS Yang TP Berry RJ Bailey LB (2012) Folate and DNA methylation: a review of molecular mechanisms and the evidence for folate’s role. Adv Nutr 3**:** 21–38. 22332098 10.3945/an.111.000992 PMC3262611

[B32] Crowley E O’Mara ML Kerr ID Callaghan R (2010) Transmembrane helix 12 plays a pivotal role in coupling energy provision and drug binding in ABCB1. FEBS J 277**:** 3974–3985. 20731718 10.1111/j.1742-4658.2010.07789.x

[B33] Damiani D Tiribelli M Calistri E Geromin A Chiarvesio A Michelutti A Cavallin M Fanin R (2006) The prognostic value of P-glycoprotein (ABCB) and breast cancer resistance protein (ABCG2) in adults with de novo acute myeloid leukemia with normal karyotype. Haematologica 91**:** 825–828. 16704962

[B34] Dang Y Zhou D Du X Zhao H Lee C-H Yang J Wang Y Qin C Guo Z Zhang Z (2022) Molecular mechanism of substrate recognition by folate transporter SLC19A1. Cell Discov 8**:** 1–11. 36575193 10.1038/s41421-022-00508-w PMC9794768

[B35] Daniels G (2022) Augustine blood group system and equilibrative nucleoside transporter 1. Transfus Med Hemother 49**:** 25–29. 35221865 10.1159/000520596 PMC8832251

[B36] Daniels G Ballif BA Helias V Saison C Grimsley S Mannessier L Hustinx H Lee E Cartron JP Peyrard T (2015) Lack of the nucleoside transporter ENT1 results in the Augustine-null blood type and ectopic mineralization. Blood 125**:** 3651–3654. 25896650 10.1182/blood-2015-03-631598 PMC4458803

[B37] Danø K (1973) Active outward transport of daunomycin in resistant Ehrlich ascites tumor cells. Biochim Biophys Acta 323**:** 466–483. 4796512 10.1016/0005-2736(73)90191-0

[B38] Dastvan R Mishra S Peskova YB Nakamoto RK Mchaourab HS (2019) Mechanism of allosteric modulation of P-glycoprotein by transport substrates and inhibitors. Science 364**:** 689–692. 31097669 10.1126/science.aav9406 PMC6890515

[B39] Davidson AL Shuman HA Nikaido H (1992) Mechanism of maltose transport in Escherichia coli: transmembrane signaling by periplasmic binding proteins. Proc Natl Acad Sci USA 89**:** 2360–2364. 1549599 10.1073/pnas.89.6.2360 PMC48657

[B40] Dean M Hamon Y Chimini G (2001) The human ATP-binding cassette (ABC) transporter superfamily. J Lipid Res 42**:** 1007–1017. 11441126

[B41] Deeley RG Cole SPC (2006) Substrate recognition and transport by multidrug resistance protein 1 (ABCC1). FEBS Lett 580**:** 1103–1111. 16387301 10.1016/j.febslet.2005.12.036

[B42] Deeley RG Westlake C Cole SPC (2006) Transmembrane transport of endo- and xenobiotics by mammalian ATP-binding cassette multidrug resistance proteins. Physiol Rev 86**:** 849–899. 16816140 10.1152/physrev.00035.2005

[B43] Dehghan A Köttgen A Yang Q Hwang SJ Kao WL Rivadeneira F Boerwinkle E Levy D Hofman A Astor BC (2008) Association of three genetic loci with uric acid concentration and risk of gout: a genome-wide association study. Lancet 372**:** 1953–1961. 18834626 10.1016/S0140-6736(08)61343-4 PMC2803340

[B44] Deng D Xu C Sun P Wu J Yan C Hu M Yan N (2014) Crystal structure of the human glucose transporter GLUT1. Nature 510**:** 121–125. 24847886 10.1038/nature13306

[B45] de Vries NA Zhao J Kroon E Buckle T Beijnen JH van Tellingen O (2007) P-glycoprotein and breast cancer resistance protein: two dominant transporters working together in limiting the brain penetration of topotecan. Clin Cancer Res 13**:** 6440–6449. 17975156 10.1158/1078-0432.CCR-07-1335

[B46] Dey S Ramachandra M Pastan I Gottesman MM Ambudkar SV (1997) Evidence for two nonidentical drug-interaction sites in the human P-glycoprotein. Proc Natl Acad Sci USA 94**:** 10594–10599. 9380680 10.1073/pnas.94.20.10594 PMC23414

[B47] Dinić J Podolski-Renić A Jeremić M Pešić M (2018) Potential of natural-based anticancer compounds for P-glycoprotein inhibition. Curr Pharm Des 24**:** 4334–4354. 30648503 10.2174/1381612825666190112164211

[B48] Dixon KH Lanpher BC Chiu J Kelley K Cowan KH (1994) A novel cDNA restores reduced folate carrier activity and methotrexate sensitivity to transport deficient cells. J Biol Chem 269**:** 17–20. 8276792

[B49] Doyle LA Yang W Abruzzo LV Krogmann T Gao Y Rishi AK Ross DD (1998) A multidrug resistance transporter from human MCF-7 breast cancer cells. Proc Natl Acad Sci USA 95**:** 15665–15670. 9861027 10.1073/pnas.95.26.15665 PMC28101

[B50] Drew D North RA Nagarathinam K Tanabe M (2021) Structures and general transport mechanisms by the major facilitator superfamily (MFS). Chem Rev 121: 5289–5335. 33886296 10.1021/acs.chemrev.0c00983 PMC8154325

[B51] Endres CJ Sengupta DJ Unadkat JD (2004) Mutation of leucine-92 selectively reduces the apparent affinity of inosine, guanosine, NBMPR [S6-(4-nitrobenzyl)-mercaptopurine riboside] and dilazep for the human equilibrative nucleoside transporter, hENT1. Biochem J 380**:** 131–137. 14759222 10.1042/BJ20031880 PMC1224139

[B52] Endres CJ Unadkat JD (2005) Residues Met89 and Ser160 in the human equilibrative nucleoside transporter 1 affect its affinity for adenosine, guanosine, S6-(4-nitrobenzyl)-mercaptopurine riboside, and dipyridamole. Mol Pharmacol 67**:** 837–844. 15557207 10.1124/mol.104.008102

[B53] Esser L Zhou F Pluchino KM Shiloach J Ma J Tang WK Gutierrez C Zhang A Shukla S Madigan JP (2017) Structures of the multidrug transporter P-glycoprotein reveal asymmetric ATP binding and the mechanism of polyspecificity. J Biol Chem 292**:** 446–461. 27864369 10.1074/jbc.M116.755884 PMC5241723

[B54] Farrell JJ Elsaleh H Garcia M Lai R Ammar A Regine WF Abrams R Benson AB Macdonald J Cass CE (2009) Human equilibrative nucleoside transporter 1 levels predict response to gemcitabine in patients with pancreatic cancer. Gastroenterology 136**:** 187–195. 18992248 10.1053/j.gastro.2008.09.067

[B55] Fetsch PA Abati A Litman T Morisaki K Honjo Y Mittal K Bates SE (2006) Localization of the ABCG2 mitoxantrone resistance-associated protein in normal tissues. Cancer Lett 235**:** 84–92. 15990223 10.1016/j.canlet.2005.04.024

[B56] FitzGerald GA (1987) Dipyridamole. N Engl J Med 316**:** 1247–1257. 3553945 10.1056/NEJM198705143162005

[B57] Ford RC Marshall-Sabey D Schuetz J (2020) Linker domains: why ABC Transporters “live in fragments no longer.” Trends Biochem Sci 45**:** 137–148. 31839525 10.1016/j.tibs.2019.11.004 PMC7219603

[B58] Fukuda Y Wang Y Lian S Lynch J Nagai S Fanshawe B Kandilci A Janke LJ Neale G Fan Y (2017) Upregulated heme biosynthesis, an exploitable vulnerability in MYCN-driven leukemogenesis. JCI Insight 2**:** e92409. 28768907 10.1172/jci.insight.92409 PMC5543914

[B59] Futamata R Ogasawara F Ichikawa T Kodan A Kimura Y Kioka N Ueda K (2020) *In vivo* FRET analyses reveal a role of ATP hydrolysis-associated conformational changes in human P-glycoprotein. J Biol Chem 295**:** 5002–5011. 32111736 10.1074/jbc.RA119.012042 PMC7152753

[B60] George AM, editor (2016) ABC Transporters—40 Years On. Springer International Publishing, Cham, Switzerland.

[B61] Goldman ID (1971) The characteristics of the membrane transport of amethopterin and the naturally occurring folates. Ann N Y Acad Sci 186**:** 400–422. 5289428 10.1111/j.1749-6632.1971.tb46996.x

[B62] Goldman ID (1969) Transport energetics of the folic acid analogue, methotrexate, in L1210 leukemia cells. Enhanced accumulation by metabolic inhibitors. J Biol Chem 244**:** 3779–3785. 5805398

[B63] Goldman ID Lichtenstein NS Oliverio VT (1968) Carrier-mediated transport of the folic acid analogue, methotrexate, in the L1210 leukemia cell. J Biol Chem 243**:** 5007–5017. 5303004

[B64] Goodell MA Brose K Paradis G Conner AS Mulligan RC (1996) Isolation and functional properties of murine hematopoietic stem cells that are replicating in vivo. J Exp Med 183**:** 1797–1806. 8666936 10.1084/jem.183.4.1797 PMC2192511

[B65] Gose T Shafi T Fukuda Y Das S Wang Y Allcock A Gavan McHarg A Lynch J Chen T Tamai I (2020) ABCG2 requires a single aromatic amino acid to “clamp” substrates and inhibitors into the binding pocket. FASEB J 34**:** 4890–4903. 32067270 10.1096/fj.201902338RR PMC8317467

[B66] Gottesman MM Fojo T Bates SE (2002) Multidrug resistance in cancer: role of ATP-dependent transporters. Nat Rev Cancer 2**:** 48–58. 11902585 10.1038/nrc706

[B67] Grant CE Gao M DeGorter MK Cole SPC Deeley RG (2008) Structural determinants of substrate specificity differences between human multidrug resistance protein (MRP) 1 (ABCC1) and MRP3 (ABCC3). Drug Metab Dispos 36**:** 2571–2581. 18775981 10.1124/dmd.108.022491

[B68] Greenhalf W Ghaneh P Neoptolemos JP Palmer DH Cox TF Lamb RF Garner E Campbell F Mackey JR Costello E ; European Study Group for Pancreatic Cancer (2014) Pancreatic cancer hENT1 expression and survival from gemcitabine in patients from the ESPAC-3 trial. J Natl Cancer Inst 106**:** djt347. 24301456 10.1093/jnci/djt347

[B69] Gyöngy Z Mocsár G Hegedűs É Stockner T Ritter Z Homolya L Schamberger A Orbán TI Remenyik J Szakacs G (2023) Nucleotide binding is the critical regulator of ABCG2 conformational transitions. eLife 12**:** e83976. 36763413 10.7554/eLife.83976 PMC9917445

[B70] Haber M Smith J Bordow SB Flemming C Cohn SL London WB Marshall GM Norris MD (2006) Association of high-level MRP1 expression with poor clinical outcome in a large prospective study of primary neuroblastoma. J Clin Oncol 24**:** 1546–1553. 16575006 10.1200/JCO.2005.01.6196

[B71] Hagmann W Jesnowski R Löhr JM (2010) Interdependence of gemcitabine treatment, transporter expression, and resistance in human pancreatic carcinoma cells. Neoplasia 12**:** 740–747. 20824050 10.1593/neo.10576 PMC2933694

[B72] Haimeur A Conseil G Deeley RG Cole SP (2004) The MRP-related and BCRP/ABCG2 multidrug resistance proteins: biology, substrate specificity and regulation. Curr Drug Metab 5**:** 21–53. 14965249 10.2174/1389200043489199

[B73] Haimeur A Deeley RG Cole SPC (2002) Charged amino acids in the sixth transmembrane helix of multidrug resistance protein 1 (MRP1/ABCC1) are critical determinants of transport activity. J Biol Chem 277**:** 41326–41333. 12186871 10.1074/jbc.M206228200

[B74] Hegedüs C Telbisz Á Hegedűs T Sarkadi B Özvegy-Laczka C (2015) Lipid regulation of the ABCB1 and ABCG2 multidrug transporters. Adv Cancer Res 125**:** 97–137. 25640268 10.1016/bs.acr.2014.10.004

[B75] Henderson GB Zevely EM (1983) Structural requirements for anion substrates of the methotrexate transport system in L1210 cells. Arch Biochem Biophys 221**:** 438–446. 6838199 10.1016/0003-9861(83)90162-5

[B76] Henriksen U Fog JU Litman T Gether U (2005) Identification of intra- and intermolecular disulfide bridges in the multidrug resistance transporter ABCG2. J Biol Chem 280**:** 36926–36934. 16107343 10.1074/jbc.M502937200

[B77] Higgins CF Gottesman MM (1992) Is the multidrug transporter a flippase? Trends Biochem Sci 17**:** 18–21. 1374941 10.1016/0968-0004(92)90419-a

[B78] Hirschi M Johnson ZL Lee SY (2017) Visualizing multistep elevator-like transitions of a nucleoside transporter. Nature 545**:** 66–70. 28424521 10.1038/nature22057 PMC5567992

[B79] Hollenstein K Dawson RJ Locher KP (2007) Structure and mechanism of ABC transporter proteins. Curr Opin Struct Biol 17**:** 412–418. 17723295 10.1016/j.sbi.2007.07.003

[B80] Honjo Y Hrycyna CA Yan QW Medina-Pérez WY Robey RW van de Laar A Litman T Dean M Bates SE (2001) Acquired mutations in the MXR/BCRP/ABCP gene alter substrate specificity in MXR/BCRP/ABCP-overexpressing cells. Cancer Res 61**:** 6635–6639. 11559526

[B81] Hou Z Matherly LH (2014) Biology of the major facilitative folate transporters SLC19A1 and SLC46A1. Curr Top Membr 73**:** 175–204. 24745983 10.1016/B978-0-12-800223-0.00004-9 PMC4185403

[B82] Iram SH Cole SPC (2011) Expression and function of human MRP1 (ABCC1) is dependent on amino acids in cytoplasmic loop 5 and its interface with nucleotide binding domain 2. J Biol Chem 286**:** 7202–7213. 21177244 10.1074/jbc.M110.166959 PMC3044977

[B83] Iram SH Cole SPC (2012) Mutation of Glu521 or Glu535 in cytoplasmic loop 5 causes differential misfolding in multiple domains of multidrug and organic anion transporter MRP1 (ABCC1). J Biol Chem 287**:** 7543–7555. 22232552 10.1074/jbc.M111.310409 PMC3293527

[B84] Ito K Olsen SL Qiu W Deeley RG Cole SPC (2001) Mutation of a single conserved tryptophan in multidrug resistance protein 1 (MRP1/ABCC1) results in loss of drug resistance and selective loss of organic anion transport. J Biol Chem 276**:** 15616–15624. 11278867 10.1074/jbc.M011246200

[B85] Ito S Ieiri I Tanabe M Suzuki A Higuchi S Otsubo K (2001) Polymorphism of the ABC transporter genes, MDR1, MRP1 and MRP2/cMOAT, in healthy Japanese subjects. Pharmacogenetics 11**:** 175–184. 11266082 10.1097/00008571-200103000-00008

[B86] Jackson SM Manolaridis I Kowal J Zechner M Taylor NMI Bause M Bauer S Bartholomaeus R Bernhardt G Koenig B Buschauer A Stahlberg H Altmann KH Locher KP (2018) Structural basis of small-molecule inhibition of human multidrug transporter ABCG2. Nat Struct Mol Bio 25**:** 333–340. 29610494 10.1038/s41594-018-0049-1

[B87] Jansen G van der Heijden J Oerlemans R Lems WF Ifergan I Scheper RJ Assaraf YG Dijkmans BAC (2004) Sulfasalazine is a potent inhibitor of the reduced folate carrier: implications for combination therapies with methotrexate in rheumatoid arthritis. Arthritis Rheum 50**:** 2130–2139. 15248210 10.1002/art.20375

[B88] Jardetzky O (1966) Simple allosteric model for membrane pumps. Nature 211**:** 969–970. 5968307 10.1038/211969a0

[B89] Jin MS Oldham ML Zhang Q Chen J (2012) Crystal structure of the multidrug transporter P-glycoprotein from Caenorhabditis elegans. Nature 490**:** 566–569. 23000902 10.1038/nature11448 PMC3482266

[B90] Johnson ZL Chen J (2018) ATP binding enables substrate release from multidrug resistance protein 1. Cell 172**:** 81–89.e10. 29290467 10.1016/j.cell.2017.12.005

[B91] Johnson ZL Chen J (2017) Structural basis of substrate recognition by the multidrug resistance protein MRP1. Cell 168**:** 1075–1085.e9. 28238471 10.1016/j.cell.2017.01.041

[B92] Jones PM George AM (2014) A reciprocating twin-channel model for ABC transporters. Q Rev Biophys 47**:** 189–220. 24786414 10.1017/S0033583514000031

[B93] Jonker JW Buitelaar M Wagenaar E Van Der Valk MA Scheffer GL Scheper RJ Plösch T Kuipers F Elferink RP Rosing H (2002) The breast cancer resistance protein protects against a major chlorophyll-derived dietary phototoxin and protoporphyria. Proc Natl Acad Sci USA 99**:** 15649–15654. 12429862 10.1073/pnas.202607599 PMC137771

[B94] Juliano RL Ling V (1976) A surface glycoprotein modulating drug permeability in Chinese hamster ovary cell mutants. Biochim Biophys Acta 455**:** 152–162. 990323 10.1016/0005-2736(76)90160-7

[B95] Kanarek N Keys HR Cantor JR Lewis CA Chan SH Kunchok T Abu-Remaileh M Freinkman E Schweitzer LD Sabatini DM (2018) Histidine catabolism is a major determinant of methotrexate sensitivity. Nature 559**:** 632–636. 29995852 10.1038/s41586-018-0316-7 PMC6082631

[B96] Kannan P Telu S Shukla S Ambudkar SV Pike VW Halldin C Gottesman MM Innis RB Hall MD (2011) The “specific” P-glycoprotein inhibitor Tariquidar is also a substrate and an inhibitor for breast cancer resistance protein (BCRP/ABCG2). ACS Chem Neurosci 2**:** 82–89. 22778859 10.1021/cn100078a PMC3369725

[B97] Karwatsky J Daoud R Cai J Gros P Georges E (2003) Binding of a photoaffinity analogue of glutathione to MRP1 (ABCC1) within two cytoplasmic regions (L0 and L1) as well as transmembrane domains 10-11 and 16-17. Biochemistry 42**:** 3286–3294. 12641460 10.1021/bi0268807

[B98] Kato S Ito K Kato Y Wakayama T Kubo Y Iseki S Tsuji A (2009) Involvement of multidrug resistance-associated protein 1 in intestinal toxicity of methotrexate. Pharm Res 26**:** 1467–1476. 19288182 10.1007/s11095-009-9858-6

[B99] Kawahara H Noguchi K Katayama K Mitsuhashi J Sugimoto Y (2010) Pharmacological interaction with sunitinib is abolished by a germ-line mutation (1291T>C) of BCRP/ABCG2 gene. Cancer Sci 101**:** 1493–1500. 20345483 10.1111/j.1349-7006.2010.01539.x PMC11158450

[B100] Khunweeraphong N Szöllősi D Stockner T Kuchler K (2019) The ABCG2 multidrug transporter is a pump gated by a valve and an extracellular lid. Nat Commun 10**:** 5433 31780715 10.1038/s41467-019-13302-2 PMC6883074

[B101] Kim Y Chen J (2018) Molecular structure of human P-glycoprotein in the ATP-bound, outward-facing conformation. Science (1979) 359**:** 915–919. 10.1126/science.aar7389 29371429

[B102] Kodan A Yamaguchi T Nakatsu T Sakiyama K Hipolito CJ Fujioka A Hirokane R Ikeguchi K Watanabe B Hiratake J (2014) Structural basis for gating mechanisms of a eukaryotic P-glycoprotein homolog. Proc Natl Acad Sci USA 111**:** 4049–4054. 24591620 10.1073/pnas.1321562111 PMC3964115

[B103] Kos V Ford RC (2009) The ATP-binding cassette family: a structural perspective. Cell Mol Life Sci 66**:** 3111–3126. 19544044 10.1007/s00018-009-0064-9 PMC11115812

[B104] Kowal J Ni D Jackson SM Manolaridis I Stahlberg H Locher KP (2021) Structural basis of drug recognition by the multidrug transporter ABCG2. J Mol Biol 433**:** 166980. 33838147 10.1016/j.jmb.2021.166980

[B105] Krishnamurthy H Gouaux E (2012) X-ray structures of LeuT in substrate-free outward-open and apo inward-open states. Nature 481**:** 469–474. 22230955 10.1038/nature10737 PMC3306218

[B106] Krishnamurthy P Ross DD Nakanishi T Bailey-Dell K Zhou S Mercer KE Sarkadi B Sorrentino BP Schuetz JD (2004) The stem cell marker Bcrp/ABCG2 enhances hypoxic cell survival through interactions with heme. J Biol Chem 279**:** 24218–24225. 15044468 10.1074/jbc.M313599200

[B107] Kühnle M Egger M Müller C Mahringer A Bernhardt G Fricker G König B Buschauer A (2009) Potent and selective inhibitors of breast cancer resistance protein (ABCG2) derived from the P-glycoprotein (ABCB1) modulator tariquidar. J Med Chem 52**:** 1190–1197. 19170519 10.1021/jm8013822

[B108] Le CA Harvey DS Aller SG (2020) Structural definition of polyspecific compensatory ligand recognition by P-glycoprotein. IUCrJ 7**:** 663–672. 10.1107/S2052252520005709 PMC7340268 32695413

[B109] Leier I Jedlitschky G Buchholz U Cole SPC Deeley RG Keppler D (1994) The MRP gene encodes an ATP-dependent export pump for leukotriene C4 and structurally related conjugates. J Biol Chem 269**:** 27807–27810. 7961706

[B110] Leonard GD Polgar O Bates SE (2002) ABC transporters and inhibitors: new targets, new agents. Curr Opin Investig Drugs 3**:** 1652–1659. 12476969

[B111] Leonard GD Fojo T Bates SE (2003) The role of ABC transporters in clinical practice. Oncologist 8**:** 411–424. 14530494 10.1634/theoncologist.8-5-411

[B112] Leslie EM Deeley RG Cole SPC (2003) Bioflavonoid stimulation of glutathione transport by the 190-kDa multidrug resistance protein 1 (MRP1). Drug Metab Dispos 31**:** 11–15. 12485947 10.1124/dmd.31.1.11

[B113] Leslie EM Deeley RG Cole SPC (2005) Multidrug resistance proteins: role of P-glycoprotein, MRP1, MRP2, and BCRP (ABCG2) in tissue defense. Toxicol Appl Pharmacol 204**:** 216–237. 15845415 10.1016/j.taap.2004.10.012

[B114] Leslie EM Létourneau IJ Deeley RG Cole SPC (2003) Functional and structural consequences of cysteine substitutions in the NH2 proximal region of the human multidrug resistance protein 1 (MRP1/ABCC1). Biochemistry 42**:** 5214–5224. 12731862 10.1021/bi027076n

[B115] Li J Jaimes KF Aller SG (2014) Refined structures of mouse P-glycoprotein. Protein Sci 23**:** 34–46. 24155053 10.1002/pro.2387 PMC3892297

[B116] Li M Mei L He C Chen H Cai X Liu Y Tian R Tian Q Song J Jiang L (2019) Extrusion pump ABCC1 was first linked with nonsyndromic hearing loss in humans by stepwise genetic analysis. Genet Med 21**:** 2744–2754. 31273342 10.1038/s41436-019-0594-y

[B117] Locher KP (2016) Mechanistic diversity in ATP-binding cassette (ABC) transporters. Nat Struct Mol Biol 23**:** 487–493. 27273632 10.1038/nsmb.3216

[B118] Loe DW Almquist KC Deeley RG Cole SPC (1996) Multidrug resistance protein (MRP)-mediated transport of leukotriene C4 and chemotherapeutic agents in membrane vesicles. Demonstration of glutathione-dependent vincristine transport. J Biol Chem 271**:** 9675–9682. 8621643 10.1074/jbc.271.16.9675

[B119] Loo TW Clarke DM (2001) Defining the drug-binding site in the human multidrug resistance P-glycoprotein using a methanethiosulfonate analog of verapamil, MTS-verapamil. J Biol Chem 276**:** 14972–14979. 11279063 10.1074/jbc.M100407200

[B120] Loo TW Clarke DM (2000) Identification of residues within the drug-binding domain of the human multidrug resistance P-glycoprotein by cysteine-scanning mutagenesis and reaction with dibromobimane. J Biol Chem 275**:** 39272–39278. 11013259 10.1074/jbc.M007741200

[B121] Loo TW Clarke DM (2002) Location of the rhodamine-binding site in the human multidrug resistance P-glycoprotein. J Biol Chem 277**:** 44332–44338. 12223492 10.1074/jbc.M208433200

[B122] Loo TW Clarke DM (2017) Attachment of a “molecular spring” restores drug-stimulated ATPase activity to P-glycoprotein lacking both Q loop glutamines. Biochem Biophys Res Commun 483**:** 366–370. 28025146 10.1016/j.bbrc.2016.12.137

[B123] Luteijn RD Zaver SA Gowen BG Wyman SK Garelis NE Onia L McWhirter SM Katibah GE Corn JE Woodward JJ (2019) SLC19A1 transports immunoreactive cyclic dinucleotides. Nature 573**:** 434–438. 31511694 10.1038/s41586-019-1553-0 PMC6785039

[B124] Maeno K Nakajima A Conseil G Rothnie A Deeley RG Cole SPC (2009) Molecular basis for reduced estrone sulfate transport and altered modulator sensitivity of transmembrane helix (TM) 6 and TM17 mutants of multidrug resistance protein 1 (ABCC1). Drug Metab Dispos 37**:** 1411–1420. 19398503 10.1124/dmd.109.026633

[B125] Manolaridis I Jackson SM Taylor NMI Kowal J Stahlberg H Locher KP (2018) Cryo-EM structures of a human ABCG2 mutant trapped in ATP-bound and substrate-bound states. Nature 563**:** 426–430. 30405239 10.1038/s41586-018-0680-3 PMC6379061

[B126] Marcé S Molina-Arcas M Villamor N Casado FJ Campo E Pastor-Anglada M Colomer D (2006) Expression of human equilibrative nucleoside transporter 1 (hENT1) and its correlation with gemcitabine uptake and cytotoxicity in mantle cell lymphoma. Haematologica 91**:** 895–902. 16818276

[B127] Maréchal R Mackey JR Lai R Demetter P Peeters M Polus M Cass CE Young J Salmon I Devière J (2009) Human equilibrative nucleoside transporter 1 and human concentrative nucleoside transporter 3 predict survival after adjuvant gemcitabine therapy in resected pancreatic adenocarcinoma. Clin Cancer Res 15**:** 2913–2919. 19318496 10.1158/1078-0432.CCR-08-2080

[B128] Matherly LH Hou Z (2008) Structure and function of the reduced folate carrier a paradigm of a major facilitator superfamily mammalian nutrient transporter. Vitam Horm 79**:** 145–184. 18804694 10.1016/S0083-6729(08)00405-6 PMC3806226

[B129] Matherly LH Hou Z Deng Y (2007) Human reduced folate carrier: translation of basic biology to cancer etiology and therapy. Cancer Metastasis Rev 26**:** 111–128. 17334909 10.1007/s10555-007-9046-2

[B130] Matherly LH Wilson MR Hou Z (2014) The major facilitative folate transporters solute carrier 19A1 and solute carrier 46A1: biology and role in antifolate chemotherapy of cancer. Drug Metab Dispos 42**:** 632–649. 24396145 10.1124/dmd.113.055723 PMC3965896

[B131] McDevitt CA Crowley E Hobbs G Starr KJ Kerr ID Callaghan R (2008) Is ATP binding responsible for initiating drug translocation by the multidrug transporter ABCG2? FEBS J 275**:** 4354–4362. 18657189 10.1111/j.1742-4658.2008.06578.x

[B132] Miyake K Mickley L Litman T Zhan Z Robey R Cristensen B Brangi M Greenberger L Dean M Fojo T (1999) Molecular cloning of cDNAs which are highly overexpressed in mitoxantrone-resistant cells: demonstration of homology to ABC transport genes. Cancer Res 59**:** 8–13. 9892175

[B133] Morfouace M Cheepala S Jackson S Fukuda Y Patel YT Fatima S Kawauchi D Shelat AA Stewart CF Sorrentino BP (2015) ABCG2 transporter expression impacts group 3 medulloblastoma response to chemotherapy. Cancer Res 75**:** 3879–3889. 26199091 10.1158/0008-5472.CAN-15-0030 PMC4573843

[B134] Moscow JA Gong M He R Sgagias MK Dixon KH Anzick SL Meltzer PS Cowan KH (1995) Isolation of a gene encoding a human reduced folate carrier (RFC1) and analysis of its expression in transport-deficient, methotrexate-resistant human breast cancer cells. Cancer Res 55**:** 3790–3794. 7641195

[B135] Mózner O Bartos Z Zámbó B Homolya L Hegedűs T Sarkadi B (2019) Cellular processing of the ABCG2 transporter-potential effects on gout and drug metabolism. Cells 8**:** 1215. 31597297 10.3390/cells8101215 PMC6830335

[B136] Nakagawa H Wakabayashi-Nakao K Tamura A Toyoda Y Koshiba S Ishikawa T (2009) Disruption of N-linked glycosylation enhances ubiquitin-mediated proteasomal degradation of the human ATP-binding cassette transporter ABCG2. FEBS J 276**:** 7237–7252. 19909340 10.1111/j.1742-4658.2009.07423.x

[B137] Nicklisch SCT Rees SD McGrath AP Gökirmak T Bonito LT Vermeer LM Cregger C Loewen G Sandin S Chang G (2016) Global marine pollutants inhibit P-glycoprotein: Environmental levels, inhibitory effects, and cocrystal structure. Sci Adv 2**:** e1600001. 27152359 10.1126/sciadv.1600001 PMC4846432

[B138] Nomura N Verdon G Kang HJ Shimamura T Nomura Y Sonoda Y Hussien SA Qureshi AA Coincon M Sato Y (2015) Structure and mechanism of the mammalian fructose transporter GLUT5. Nature 526**:** 397–401. 26416735 10.1038/nature14909 PMC4618315

[B139] Nosol K Romane K Irobalieva RN Alam A Kowal J Fujita N Locher KP (2020) Cryo-EM structures reveal distinct mechanisms of inhibition of the human multidrug transporter ABCB1. Proc Natl Acad Sci USA 117**:** 26245–26253. 33020312 10.1073/pnas.2010264117 PMC7585025

[B140] O’Connor C Wallace-Povirk A Ning C Frühauf J Tong N Gangjee A Matherly LH Hou Z (2021) Folate transporter dynamics and therapy with classic and tumor-targeted antifolates. Sci Rep 11**:** 6389. 33737637 10.1038/s41598-021-85818-x PMC7973545

[B141] Oldham ML Chen J (2011a) Crystal structure of the maltose transporter in a pretranslocation intermediate state. Science 332**:** 1202–1205. 21566157 10.1126/science.1200767

[B142] Oldham ML Chen J (2011b) Snapshots of the maltose transporter during ATP hydrolysis. Proc Natl Acad Sci USA 108**:** 15152–15156. 21825153 10.1073/pnas.1108858108 PMC3174604

[B143] Oldham ML Khare D Quiocho FA Davidson AL Chen J (2007) Crystal structure of a catalytic intermediate of the maltose transporter. Nature 450**:** 515–521. 18033289 10.1038/nature06264

[B144] Orlando BJ Liao M (2020) ABCG2 transports anticancer drugs via a closed-to-open switch. Nat Commun 11**:** 2264. 32385283 10.1038/s41467-020-16155-2 PMC7210939

[B145] Oselin K Mrozikiewicz PM Gaikovitch E Pähkla R Roots I (2003) Frequency of MRP1 genetic polymorphisms and their functional significance in Caucasians: detection of a novel mutation G816A in the human MRP1 gene. Eur J Clin Pharmacol 59**:** 347–350. 12856092 10.1007/s00228-003-0625-z

[B146] Pajic M Murray J Marshall GM Cole SPC Norris MD Haber M (2011) ABCC1 G2012T single nucleotide polymorphism is associated with patient outcome in primary neuroblastoma and altered stability of the ABCC1 gene transcript. Pharmacogenet Genomics 21**:** 270–279. 21317832 10.1097/FPC.0b013e328343dd5f

[B147] Paproski RJ Visser F Zhang J Tackaberry T Damaraju V Baldwin SA Young JD Cass CE (2008) Mutation of Trp29 of human equilibrative nucleoside transporter 1 alters affinity for coronary vasodilator drugs and nucleoside selectivity. Biochem J 414**:** 291–300. 18462193 10.1042/BJ20080074

[B148] Pastor-Anglada M Mata-Ventosa A Pérez-Torras S (2022) Inborn errors of nucleoside transporter (NT)-encoding genes (*SLC28* and *SLC29*). Int J Mol Sci 23**:** 8770. 35955904 10.3390/ijms23158770 PMC9369021

[B149] Paulusma CC van Geer MA Evers R Heijn M Ottenhoff R Borst P Oude Elferink RPJ (1999) Canalicular multispecific organic anion transporter/multidrug resistance protein 2 mediates low-affinity transport of reduced glutathione. Biochem J 338**:** 393–401. 10024515 PMC1220065

[B150] Payen LF Gao M Westlake CJ Cole SPC Deeley RG (2003) Role of carboxylate residues adjacent to the conserved core Walker B motifs in the catalytic cycle of multidrug resistance protein 1 (ABCC1). J Biol Chem 278**:** 38537–38547. 12882957 10.1074/jbc.M305786200

[B151] Penmatsa A Wang KH Gouaux E (2013) X-ray structure of dopamine transporter elucidates antidepressant mechanism. Nature 503**:** 85–90. 24037379 10.1038/nature12533 PMC3904663

[B152] Perdu J Germain DP (2001) Identification of novel polymorphisms in the pM5 and MRP1 (ABCC1) genes at locus 16p13.1 and exclusion of both genes as responsible for pseudoxanthoma elasticum. Hum Mutat 17**:** 74–75. 10.1002/1098-1004(2001)17:1<74::AID-HUMU14>3.0.CO;2-F 11139250

[B153] Pham AN Wang J Fang J Gao X Zhang Y Blower PE Sadée W Huang Y (2009) Pharmacogenomics approach reveals MRP1 (ABCC1)-mediated resistance to geldanamycins. Pharm Res 26**:** 936–945. 19067123 10.1007/s11095-008-9796-8

[B154] Pilotto Heming C Muriithi W Wanjiku Macharia L Niemeyer Filho P Moura-Neto V Aran V (2022) P-glycoprotein and cancer: what do we currently know? Heliyon 8**:** e11171. 36325145 10.1016/j.heliyon.2022.e11171 PMC9618987

[B155] Pizzagalli MD Bensimon A Superti-Furga G (2021) A guide to plasma membrane solute carrier proteins. FEBS J 288**:** 2784–2835. 32810346 10.1111/febs.15531 PMC8246967

[B156] Prasad PD Ramamoorthy S Leibach FH Ganapathy V (1995) Molecular cloning of the human placental folate transporter. Biochem Biophys Res Commun 206**:** 681–687. 7826387 10.1006/bbrc.1995.1096

[B157] Quistgaard EM Löw C Guettou F Nordlund P (2016) Understanding transport by the major facilitator superfamily (MFS): structures pave the way. Nat Rev Mol Cell Biol 17**:** 123–132. 26758938 10.1038/nrm.2015.25

[B158] Qureshi AA Suades A Matsuoka R Brock J McComas SE Nji E Orellana L Claesson M Delemotte L Drew D (2020) The molecular basis for sugar import in malaria parasites. Nature 578**:** 321–325. 31996846 10.1038/s41586-020-1963-z

[B159] Rasouli A Yu Q Dehghani-Ghahnaviyeh S Wen PC Kowal J Locher KP Tajkhorshid E (2023) Differential dynamics and direct interaction of bound ligands with lipids in multidrug transporter ABCG2. Proc Natl Acad Sci USA 120**:** e2213437120. 36580587 10.1073/pnas.2213437120 PMC9910490

[B160] Raviv Y Pollard HB Bruggemann EP Pastan I Gottesman MM (1990) Photosensitized labeling of a functional multidrug transporter in living drug-resistant tumor cells. J Biol Chem 265**:** 3975–3980. 1968065

[B161] Rees DC Johnson E Lewinson O (2009) ABC transporters: the power to change. Nat Rev Mol Cell Biol 10**:** 218–227. 19234479 10.1038/nrm2646 PMC2830722

[B162] Renes J de Vries EGE Nienhuis EF Jansen PLM Müller M (1999) ATP- and glutathione-dependent transport of chemotherapeutic drugs by the multidrug resistance protein MRP1. Br J Pharmacol 126**:** 681–688. 10188979 10.1038/sj.bjp.0702360 PMC1565864

[B163] Ricci JW Lovato D Larson RS (2015) ABCG2 inhibitors: will they find clinical relevance? J Dev Drugs 4**:** 138.

[B164] Ritchie C Cordova AF Hess GT Bassik MC Li L (2019) SLC19A1 is an importer of the immunotransmitter cGAMP. Mol Cell 75**:** 372–381.e5. 31126740 10.1016/j.molcel.2019.05.006 PMC6711396

[B165] Roninson IB Chin JE Choi KG Gros P Housman DE Fojo A Shen DW Gottesman MM Pastan I (1986) Isolation of human mdr DNA sequences amplified in multidrug-resistant KB carcinoma cells. Proc Natl Acad Sci USA 83**:** 4538–4542. 3459187 10.1073/pnas.83.12.4538 PMC323769

[B166] Saito S Iida A Sekine A Miura Y Ogawa C Kawauchi S Higuchi S Nakamura Y (2002) Identification of 779 genetic variations in eight genes encoding members of the ATP-binding cassette, subfamily C (ABCC/MRP/CFTR. J Hum Genet 47**:** 147–171. 12166651 10.1007/s100380200018

[B167] Sajid A Lusvarghi S Ambudkar SV (2022) The P‐glycoprotein multidrug transporter, in Drug Transporters (You G Morris ME, eds) pp 199–211., Wiley, Hoboken, NJ.

[B168] Salerno M Garnier-Suillerot A (2001) Kinetics of glutathione and daunorubicin efflux from multidrug resistance protein overexpressing small-cell lung cancer cells. Eur J Pharmacol 421**:** 1–9. 11408043 10.1016/s0014-2999(01)00992-x

[B169] Sasabe H Kato Y Suzuki T Itose M Miyamoto G Sugiyama Y (2004) Differential involvement of multidrug resistance-associated protein 1 and P-glycoprotein in tissue distribution and excretion of grepafloxacin in mice. J Pharmacol Exp Ther 310**:** 648–655. 15131241 10.1124/jpet.104.065201

[B170] Schinkel AH Smit JJM van Tellingen O Beijnen JH Wagenaar E van Deemter L Mol CAAM van der Valk MA Robanus-Maandag EC te Riele HPJ (1994) Disruption of the mouse mdr1a P-glycoprotein gene leads to a deficiency in the blood-brain barrier and to increased sensitivity to drugs. Cell 77**:** 491–502. 7910522 10.1016/0092-8674(94)90212-7

[B171] Seelig A Blatter XL Wohnsland F (2000) Substrate recognition by P-glycoprotein and the multidrug resistance-associated protein MRP1: a comparison. Int J Clin Pharmacol Ther 38**:** 111–121. 10739114 10.5414/cpp38111

[B172] Semsei AF Erdelyi DJ Ungvari I Csagoly E Hegyi MZ Kiszel PS Lautner-Csorba O Szabolcs J Masat P Fekete G (2012) ABCC1 polymorphisms in anthracycline-induced cardiotoxicity in childhood acute lymphoblastic leukaemia. Cell Biol Int 36**:** 79–86. 21929509 10.1042/CBI20110264

[B173] SenGupta DJ Unadkat JD (2004) Glycine 154 of the equilibrative nucleoside transporter, hENT1, is important for nucleoside transport and for conferring sensitivity to the inhibitors nitrobenzylthioinosine, dipyridamole, and dilazep. Biochem Pharmacol 67**:** 453–458. 15037197 10.1016/j.bcp.2003.09.018

[B174] Shayeghi M Latunde-Dada GO Oakhill JS Laftah AH Takeuchi K Halliday N Khan Y Warley A McCann FE Hider RC (2005) Identification of an intestinal heme transporter. Cell 122**:** 789–801. 16143108 10.1016/j.cell.2005.06.025

[B175] Siedlinski M Boezen HM Boer JM Smit HA Postma DS (2009) ABCC1 polymorphisms contribute to level and decline of lung function in two population-based cohorts. Pharmacogenet Genomics 19**:** 675–684. 19687781 10.1097/FPC.0b013e32832f5eff

[B176] Sissung TM Baum CE Kirkland CT Gao R Gardner ER Figg WD (2010) Pharmacogenetics of membrane transporters: an update on current approaches. Mol Biotechnol 44**:** 152–167. 19950006 10.1007/s12033-009-9220-6 PMC6362991

[B177] Slot AJ Molinski SV Cole SP (2011) Mammalian multidrug-resistance proteins (MRPs). Essays Biochem 50**:** 179–207. 21967058 10.1042/bse0500179

[B178] Spratlin J Sangha R Glubrecht D Dabbagh L Young JD Dumontet C Cass C Lai R Mackey JR (2004) The absence of human equilibrative nucleoside transporter 1 is associated with reduced survival in patients with gemcitabine-treated pancreas adenocarcinoma. Clin Cancer Res 10**:** 6956–6961. 15501974 10.1158/1078-0432.CCR-04-0224

[B179] Stockner T Gradisch R Schmitt L (2020) The role of the degenerate nucleotide binding site in type I ABC exporters. FEBS Lett 594**:** 3815–3838. 33179257 10.1002/1873-3468.13997 PMC7756269

[B180] Svaton M Skvarova Kramarzova K Kanderova V Mancikova A Smisek P Jesina P Krijt J Stiburkova B Dobrovolny R Sokolova J (2020) A homozygous deletion in the SLC19A1 gene as a cause of folate-dependent recurrent megaloblastic anemia. Blood 135**:** 2427–2431. 32276275 10.1182/blood.2019003178 PMC7330012

[B181] Szakács G Paterson JK Ludwig JA Booth-Genthe C Gottesman MM (2006) Targeting multidrug resistance in cancer. Nat Rev Drug Discov 5**:** 219–234. 16518375 10.1038/nrd1984

[B182] Taguchi Y Saeki K Komano T (2002) Functional analysis of MRP1 cloned from bovine. FEBS Lett 521**:** 211–213. 12067707 10.1016/s0014-5793(02)02816-8

[B183] Tanaka M Javle M Dong X Eng C Abbruzzese JL Li D (2010) Gemcitabine metabolic and transporter gene polymorphisms are associated with drug toxicity and efficacy in patients with locally advanced pancreatic cancer. Cancer 116**:** 5325–5335. 20665488 10.1002/cncr.25282 PMC2966859

[B184] Taylor NMI Manolaridis I Jackson SM Kowal J Stahlberg H Locher KP (2017) Structure of the human multidrug transporter ABCG2. Nature 546**:** 504–509. 28554189 10.1038/nature22345

[B185] Telbisz Á Özvegy-Laczka C Hegedűs T Váradi A Sarkadi B (2013) Effects of the lipid environment, cholesterol and bile acids on the function of the purified and reconstituted human ABCG2 protein. Biochem J 450**:** 387–395. 23205634 10.1042/BJ20121485

[B186] ten Hove T Drillenburg P Wijnholds J Te Velde AA van Deventer SJH (2002) Differential susceptibility of multidrug resistance protein-1 deficient mice to DSS and TNBS-induced colitis. Dig Dis Sci 47**:** 2056–2063. 12353855 10.1023/a:1019629013945

[B187] Ueda K Cornwell MM Gottesman MM Pastan I Roninson IB Ling V Riordan JR (1986) The mdr1 gene, responsible for multidrug-resistance, codes for P-glycoprotein. Biochem Biophys Res Commun 141**:** 956–962. 2880583 10.1016/s0006-291x(86)80136-x

[B188] Urgaonkar S Nosol K Said AM Nasief NN Bu Y Locher KP Lau JYN Smolinski MP (2022) Discovery and characterization of potent dual P-glycoprotein and CYP3A4 inhibitors: design, synthesis, cryo-EM analysis, and biological evaluations. J Med Chem 65**:** 191–216. 34928144 10.1021/acs.jmedchem.1c01272

[B189] US Food and Drug Administration (2017) In Vitro Metabolism- and Transporter-Mediated Drug-Drug: Interaction Studies Guidance for Industry. Center for Drug Evaluation and Research, Washington, DC.

[B190] Vahedi S Lusvarghi S Pluchino K Shafrir Y Durell SR Gottesman MM Ambudkar S V. (2018) Mapping discontinuous epitopes for MRK-16, UIC2 and 4E3 antibodies to extracellular loops 1 and 4 of human P-glycoprotein. Sci Rep 8**:** 1–13. 30143707 10.1038/s41598-018-30984-8 PMC6109178

[B191] Verhalen B Dastvan R Thangapandian S Peskova Y Koteiche HA Nakamoto RK Tajkhorshid E Mchaourab HS (2017) Energy transduction and alternating access of the mammalian ABC transporter P-glycoprotein. Nature 543**:** 738–741. 28289287 10.1038/nature21414 PMC5558441

[B192] Visser F Baldwin SA Isaac RE Young JD Cass CE (2005) Identification and mutational analysis of amino acid residues involved in dipyridamole interactions with human and Caenorhabditis elegans equilibrative nucleoside transporters. J Biol Chem 280**:** 11025–11034. 15649894 10.1074/jbc.M410348200

[B193] Visser F Sun L Damaraju V Tackaberry T Peng Y Robins MJ Baldwin SA Young JD Cass CE (2007) Residues 334 and 338 in transmembrane segment 8 of human equilibrative nucleoside transporter 1 are important determinants of inhibitor sensitivity, protein folding, and catalytic turnover. J Biol Chem 282**:** 14148–14157. 17379602 10.1074/jbc.M701735200

[B194] Visser F Vickers MF Ng AML Baldwin SA Young JD Cass CE (2002) Mutation of residue 33 of human equilibrative nucleoside transporters 1 and 2 alters sensitivity to inhibition of transport by dilazep and dipyridamole. J Biol Chem 277**:** 395–401. 11689555 10.1074/jbc.M105324200

[B195] Visser F Zhang J Raborn RT Baldwin SA Young JD Cass CE (2005) Residue 33 of human equilibrative nucleoside transporter 2 is a functionally important component of both the dipyridamole and nucleoside binding sites. Mol Pharmacol 67**:** 1291–1298. 15644498 10.1124/mol.104.005884

[B196] Voutsadakis IA (2011) Molecular predictors of gemcitabine response in pancreatic cancer. World J Gastrointest Oncol 3**:** 153–164. 22110842 10.4251/wjgo.v3.i11.153 PMC3220724

[B197] Wang J Gan C Sparidans RW Wagenaar E van Hoppe S Beijnen JH Schinkel AH (2018) P-glycoprotein (MDR1/ABCB1) and Breast Cancer Resistance Protein (BCRP/ABCG2) affect brain accumulation and intestinal disposition of encorafenib in mice. Pharmacol Res 129**:** 414–423. 29155017 10.1016/j.phrs.2017.11.006

[B198] Wang L Johnson ZL Wasserman MR Levring J Chen J Liu S (2020) Characterization of the kinetic cycle of an ABC transporter by single-molecule and cryo-EM analyses. eLife 9**:** 1–20. 10.7554/eLife.56451 PMC7253176 32458799

[B199] Wang P Sachar M Lu J Shehu AI Zhu J Chen J Liu K Anderson KE Xie W Gonzalez FJ (2019) The essential role of the transporter ABCG2 in the pathophysiology of erythropoietic protoporphyria. Sci Adv 5**:** eaaw6127 31555729 10.1126/sciadv.aaw6127 PMC6750912

[B200] Ward AB Szewczyk P Grimard V Lee CW Martinez L Doshi R Caya A Villaluz M Pardon E Cregger C (2013) Structures of P-glycoprotein reveal its conformational flexibility and an epitope on the nucleotide-binding domain. Proc Natl Acad Sci USA 110**:** 13386–13391. 23901103 10.1073/pnas.1309275110 PMC3746859

[B201] Wen PC Verhalen B Wilkens S Mchaourab HS Tajkhorshid E (2013) On the origin of large flexibility of P-glycoprotein in the inward-facing state. J Biol Chem 288**:** 19211–19220. 23658020 10.1074/jbc.M113.450114 PMC3696692

[B202] Westlake CJ Qian YM Gao M Vasa M Cole SPC Deeley RG (2003) Identification of the structural and functional boundaries of the multidrug resistance protein 1 cytoplasmic loop 3. Biochemistry 42**:** 14099–14113. 14640677 10.1021/bi035333y

[B203] Wijnholds J Evers R van Leusden MR Mol CAAM Zaman GJR Mayer U Beijnen JH van der Valk M Krimpenfort P Borst P (1997) Increased sensitivity to anticancer drugs and decreased inflammatory response in mice lacking the multidrug resistance-associated protein. Nat Med 3**:** 1275–1279. 9359705 10.1038/nm1197-1275

[B204] Wijnholds J Scheffer GL van der Valk M van der Valk P Beijnen JH Scheper RJ Borst P (1998) Multidrug resistance protein 1 protects the oropharyngeal mucosal layer and the testicular tubules against drug-induced damage. J Exp Med 188**:** 797–808. 9730882 10.1084/jem.188.5.797 PMC2213389

[B205] Wilkens S (2015) Structure and mechanism of ABC transporters. F1000Prime Rep 7**:** 14. 25750732 10.12703/P7-14 PMC4338842

[B206] Williams FM Murray RC Underhill TM Flintoff WF (1994) Isolation of a hamster cDNA clone coding for a function involved in methotrexate uptake. J Biol Chem 269**:** 5810–5816. 8119923

[B207] Williams FMR Flintoff WF (1995) Isolation of a human cDNA that complements a mutant hamster cell defective in methotrexate uptake. J Biol Chem 270**:** 2987–2992. 7852378 10.1074/jbc.270.7.2987

[B208] Wong SC Proefke SA Bhushan A Matherly LH (1995) Isolation of human cDNAs that restore methotrexate sensitivity and reduced folate carrier activity in methotrexate transport-defective Chinese hamster ovary cells. J Biol Chem 270**:** 17468–17475. 7615551 10.1074/jbc.270.29.17468

[B209] Woodahl EL Wang J Heimfeld S Ren AG McCune JS (2008) Imatinib inhibition of fludarabine uptake in T-lymphocytes. Cancer Chemother Pharmacol 62**:** 735–739. 18004569 10.1007/s00280-007-0629-y

[B210] Wright NJ Fedor JG Zhang H Jeong P Suo Y Yoo J Hong J Im W Lee SY (2022) Methotrexate recognition by the human reduced folate carrier SLC19A1. Nature 609**:** 1056–1062. 36071163 10.1038/s41586-022-05168-0 PMC9822521

[B211] Wright NJ Lee SY (2022) Recent advances on the inhibition of human solute carriers: Therapeutic implications and mechanistic insights. Curr Opin Struct Biol 74**:** 102378 35487145 10.1016/j.sbi.2022.102378 PMC9763051

[B212] Wright NJ Lee SY (2019) Structures of human ENT1 in complex with adenosine reuptake inhibitors. Nat Struct Mol Biol 26**:** 599–606. 31235912 10.1038/s41594-019-0245-7 PMC6705415

[B213] Wright NJ Lee SY (2021) Toward a molecular basis of cellular nucleoside transport in humans. Chem Rev 121**:** 5336–5358. 33232132 10.1021/acs.chemrev.0c00644 PMC10032034

[B214] Wu P Oleschuk CJ Mao Q Keller BO Deeley RG Cole SPC (2005) Analysis of human multidrug resistance protein 1 (ABCC1) by matrix-assisted laser desorption ionization/time of flight mass spectrometry: toward identification of leukotriene C4 binding sites. Mol Pharmacol 68**:** 1455–1465. 16105987 10.1124/mol.105.016576

[B215] Yang R Sowers R Mazza B Healey JH Huvos A Grier H Bernstein M Beardsley GP Krailo MD Devidas M (2003) Sequence alterations in the reduced folate carrier are observed in osteosarcoma tumor samples. Clin Cancer Res 9**:** 837–844. 12576457

[B216] Yee SW Gong L Badagnani I Giacomini KM Klein TE Altman RB (2010) SLC19A1 pharmacogenomics summary. Pharmacogenet Genomics 20**:** 708–715. 20811316 10.1097/FPC.0b013e32833eca92 PMC2956130

[B217] Yoshida H Kanatsu K Muso E Kuwahara T Sekita K Ono T Konishi N Kanda C Minakata T Morimoto K (1994) Effects of an anti-platelet drug (dilazep) in IgA nephropathy: comparison of clinical effects with renal biopsy findings. Nippon Jinzo Gakkai Shi 36**:** 339–344. 8022106

[B218] Yoshioka S Katayama K Okawa C Takahashi S Tsukahara S Mitsuhashi J Sugimoto Y (2007) The identification of two germ-line mutations in the human breast cancer resistance protein gene that result in the expression of a low/non-functional protein. Pharm Res 24**:** 1108–1117. 17373578 10.1007/s11095-007-9235-2

[B219] Young JD Yao SYM Baldwin JM Cass CE Baldwin SA (2013) The human concentrative and equilibrative nucleoside transporter families, SLC28 and SLC29. Mol Aspects Med 34**:** 529–547. 23506887 10.1016/j.mam.2012.05.007

[B220] Young JD Yao SYM Sun L Cass CE Baldwin SA (2008) Human equilibrative nucleoside transporter (ENT) family of nucleoside and nucleobase transporter proteins. Xenobiotica 38**:** 995–1021. 18668437 10.1080/00498250801927427

[B221] Yu Q Ni D Kowal J Manolaridis I Jackson SM Stahlberg H Locher KP (2021) Structures of ABCG2 under turnover conditions reveal a key step in the drug transport mechanism. Nat Commun 12**:** 4376 34282134 10.1038/s41467-021-24651-2 PMC8289821

[B222] Zhang DW Nunoya K Vasa M Gu HM Cole SPC Deeley RG (2006) Mutational analysis of polar amino acid residues within predicted transmembrane helices 10 and 16 of multidrug resistance protein 1 (ABCC1): effect on substrate specificity. Drug Metab Dispos 34**:** 539–546. 16415113 10.1124/dmd.105.007740

[B223] Zhang Q Zhang X Zhu Y Sun P Zhang L Ma J Zhang Y Zeng L Nie X Gao Y (2022) Recognition of cyclic dinucleotides and folates by human SLC19A1. Nature 612**:** 170–176. 36265513 10.1038/s41586-022-05452-z

[B224] Zhao R Gao F Goldman ID (2002) Reduced folate carrier transports thiamine monophosphate: an alternative route for thiamine delivery into mammalian cells. Am J Physiol Cell Physiol 282**:** C1512–C1517. 11997266 10.1152/ajpcell.00547.2001

[B225] Zhao R Gao F Wang Y Diaz GA Gelb BD Goldman ID (2001) Impact of the reduced folate carrier on the accumulation of active thiamin metabolites in murine leukemia cells. J Biol Chem 276**:** 1114–1118. 11038362 10.1074/jbc.M007919200

[B226] Zhao R Goldman ID (2013) Folate and thiamine transporters mediated by facilitative carriers (SLC19A1-3 and SLC46A1) and folate receptors. Mol Aspects Med 34**:** 373–385. 23506878 10.1016/j.mam.2012.07.006 PMC3831518

[B227] Zhao R Goldman ID (2003) Resistance to antifolates. Oncogene 22**:** 7431–7457. 14576850 10.1038/sj.onc.1206946

[B228] Zhao R Matherly LH Goldman ID (2009) Membrane transporters and folate homeostasis: intestinal absorption and transport into systemic compartments and tissues. Expert Rev Mol Med 11**:** e4 19173758 10.1017/S1462399409000969 PMC3770294

[B229] Zhao R Russell RG Wang Y Liu L Gao F Kneitz B Edelmann W Goldman ID (2001) Rescue of embryonic lethality in reduced folate carrier-deficient mice by maternal folic acid supplementation reveals early neonatal failure of hematopoietic organs. J Biol Chem 276**:** 10224–10228. 11266438 10.1074/jbc.c000905200

[B230] Zhou S Morris JJ Barnes Y Lan L Schuetz JD Sorrentino BP (2002) Bcrp1 gene expression is required for normal numbers of side population stem cells in mice, and confers relative protection to mitoxantrone in hematopoietic cells in vivo. Proc Natl Acad Sci USA 99**:** 12339–12344. 12218177 10.1073/pnas.192276999 PMC129446

[B231] Zhou S Schuetz JD Bunting KD Colapietro A-M Sampath J Morris JJ Lagutina I Grosveld GC Osawa M Nakauchi H (2001) The ABC transporter Bcrp1/ABCG2 is expressed in a wide variety of stem cells and is a molecular determinant of the side-population phenotype. Nat Med 7**:** 1028–1034. 11533706 10.1038/nm0901-1028

[B232] Zhou S-F Wang L-L Di YM Xue CC Duan W Li CG Li Y (2008) Substrates and inhibitors of human multidrug resistance associated proteins and the implications in drug development. Curr Med Chem 15**:** 1981–2039. 18691054 10.2174/092986708785132870

[B233] Zolnerciks JK Akkaya BG Snippe M Chiba P Seelig A Linton KJ (2014) The Q loops of the human multidrug resistance transporter ABCB1 are necessary to couple drug binding to the ATP catalytic cycle. FASEB J 28**:** 4335–4346. 25016028 10.1096/fj.13-245639

